# Regulatory mechanism of trichothecene biosynthesis in *Fusarium graminearum*

**DOI:** 10.3389/fmicb.2023.1148771

**Published:** 2023-04-17

**Authors:** Maydelene Xiao Xuan Liew, Yuichi Nakajima, Kazuyuki Maeda, Naotsugu Kitamura, Makoto Kimura

**Affiliations:** Department of Applied Biosciences, Graduate School of Bioagricultural Sciences, Nagoya University, Nagoya, Aichi, Japan

**Keywords:** complex media and defined media (synthetic media), nitrogen regulatory gene AreA, structural changes of the secondary metabolite gene cluster core region, trichothecene mycotoxin, *Tri6* transcription under two types of regulation, *WOR1* ortholog *Fgp1*, zinc finger transcription factor

## Abstract

Among the genes involved in the biosynthesis of trichothecene (*Tri* genes), *Tri6* and *Tri10* encode a transcription factor with unique Cys_2_His_2_ zinc finger domains and a regulatory protein with no consensus DNA-binding sequences, respectively. Although various chemical factors, such as nitrogen nutrients, medium pH, and certain oligosaccharides, are known to influence trichothecene biosynthesis in *Fusarium graminearum*, the transcriptional regulatory mechanism of *Tri6* and *Tri10* genes is poorly understood. Particularly, culture medium pH is a major regulator in trichothecene biosynthesis in *F. graminearum*, but it is susceptible to metabolic changes posed by nutritional and genetic factors. Hence, appropriate precautions should be considered to minimize the indirect influence of pH on the secondary metabolism while studying the roles of nutritional and genetic factors on trichothecene biosynthesis regulation. Additionally, it is noteworthy that the structural changes of the trichothecene gene cluster core region exert considerable influence over the normal regulation of *Tri* gene expression. In this perspective paper, we consider a revision of our current understanding of the regulatory mechanism of trichothecene biosynthesis in *F. graminearum* and share our idea toward establishing a regulatory model of *Tri6* and *Tri10* transcription.

## Introduction

*Fusarium graminearum* and related species cause Fusarium head blight (FHB), a devastating disease of small grain cereals, such as wheat and barley (Goswami and Kistler, 2004). In addition to the yield loss, they accumulate trichothecene mycotoxins in the maturing seeds, thereby posing serious threats to the safety of grains and their commodities. As deoxynivalenol (DON) and its acetylated precursors, 3-acetyldeoxynivalenol and 15-acetyldeoxynivalenol (15-ADON), are the most prevalent trichothecenes associated with FHB, permissible limits were set for DON level in foods and feeds by administrative organizations in many countries. For the development of appropriate measures to reduce the risk of mycotoxin contamination, understanding the transcriptional regulatory mechanism of trichothecene biosynthesis genes (*Tri* genes) is important. Trichothecene biosynthesis in *F. graminearum* is known to be regulated by various nutritional and environmental factors, including the pH of the culture media (Gardiner et al., 2009b), water activity (Ramirez et al., 2006), nitrogen source (Min et al., 2012; Giese et al., 2013), oligosaccharide inducers containing an α-(1 → 2) (glucosyl/xylosyl)-fructosyl linkage (Nakajima et al., 2016), and oxidative stress (Ponts et al., 2006).

Most genes involved in trichothecene biosynthesis assemble in the gene cluster, of which the core region is comprised of four *Tri* genes, *Tri4*–*Tri6*–*Tri5*–*Tri10*, that are sufficient to build up the toxic trichothecene skeleton of isotrichodermol (Kimura et al., 2007; Desjardins, 2009; McCormick et al., 2013). Among the four minimal *Tri* genes, *Tri6* and *Tri10* are regulatory genes. *Tri6* encodes a transcription factor with three unique Cys_2_His_2_ zinc finger motifs for DNA binding to the minimum sequence of YNAGGCC (Hohn et al., 1999), and the protein is indispensable for the transcription of pathway *Tri* genes (Proctor et al., 1995). *Tri10* encodes a protein without any known DNA-binding motifs, which plays a pivotal role in the transcriptional activation of *Tri* genes (Tag et al., 2001). The remaining two genes, *Tri5* and *Tri4*, are required for the cyclization of farnesyl pyrophosphate to trichodiene (Hohn and Beremand, 1989) and oxygenations of trichodiene to isotrichotriol (McCormick et al., 2006; Tokai et al., 2007), respectively; of which the isotrichotriol is subsequently cyclized to isotrichodermol non-enzymatically under acidic conditions.

The molecular genetic study of trichothecene biosynthesis was initially started in the late 1980s by the research group of USDA-ARS (United States Department of Agriculture, Agricultural Research Service, Peoria, IL) using the T-2 toxin-producing *Fusarium sporotrichioides* NRRL 3299 as a model strain (Desjardins et al., 1993). Distinct from other *Fusarium* species such as *F. graminearum* and *F. venenatum*, the trichothecene biosynthesis is not tightly regulated in *F. sporotrichioides*. Indeed, T-2 toxin accumulates in various media under different culture conditions where *F. graminearum* fails to produce trichothecenes. With increasing availability of genomic information and molecular tools in the 2000s, research interest was shifted to the regulatory mechanism of trichothecene biosynthesis in *F. graminearum*, which is the primary causal agent of FHB (Merhej et al., 2011). Up to now, several regulatory models of *Tri* gene expression have been proposed in previous studies (Seong et al., 2009; Nasmith et al., 2011; Hou et al., 2015; Jiang et al., 2016), but a definitive model remains to be established.

While interpreting the influence of a certain nutritional or genetic factor on a biological phenotype, a “cause-and-effect” relationship should not be established unless other factors that have critical impacts on the phenotype are consistent with the control condition. Therefore, it is important to set up experimental designs so that the “effect” is not determined by such critical factors. For example, to elucidate the genetic basis of trichothecene biosynthesis regulation in *F. graminearum*, each mutant should be evaluated considering the pH, which is a major regulatory factor, to be essentially the same as that of the wild-type (Nakajima et al., 2020). In this perspective paper, we discuss the underlying issues associated with the use of various culture systems and exemplify the possible approaches to cope with the challenges. The overlooked impact of perturbing the structure of the trichothecene gene cluster core region is also highlighted, as well as the future direction of studies from methodological and strategic aspects.

## Choice of appropriate culture systems and conditions is essential for the study of the regulatory mechanism of trichothecene biosynthesis in *F. graminearum*

The pH of a medium is critical and has a decisive influence on the regulation of the secondary metabolism. Exposure to an extremely low pH bypasses the requirement of certain factors that are involved in the activation of trichothecene biosynthesis in *F. graminearum*. In addition to chemical factors, less well known are physical factors that mediate trichothecene biosynthesis. Mycelia within a submerged shake culture exist either in dispersed or aggregate forms under a certain physical condition (determined by multiple factors; e.g., culture volume, vessel size and shape, and agitation speed), which affects trichothecene biosynthesis regulation (Nakajima et al., 2014). Furthermore, the trichothecene induction is associated with mycelial morphology. For example, *F. graminearum* produces trichothecenes in aerial hyphae grown on YG medium (0.5% [w/v] yeast extract, 2% [w/v] glucose) solidified by agar, despite its inability to produce trichothecenes in submerged hyphae on liquid YG medium (Ochiai et al., 2007). Other culture conditions or components, such as salt concentrations, may also exert influence over the expression of *Tri* genes, and thus, subsequent trichothecene biosynthesis. However, on liquid media with gyratory shaking, small increases in salt concentration, needed for pH adjustments of the culture to assess the impact of agmatine (Nakajima et al., 2020), marginally affected toxin production. In this section, we show the importance of choosing appropriate culture systems and conditions for the study of the regulatory mechanism of trichothecene biosynthesis, while highlighting instances in which erroneous conclusions tend to be made and/or obvious facts could be overlooked.

### Advantages and disadvantages associated with the use of various types of culture media

Numerous dispensable and indispensable factors are involved in the induction of trichothecene biosynthesis, and their combined effects determine the amount of the mycotoxin accumulation in *F. graminearum*. Both complex and defined media (synthetic media) have been used to evaluate the role of a certain factor on trichothecene induction.

The advantage of using complex media for trichothecene biosynthesis regulatory studies is the relatively mild acidic conditions during culture and generally strong induction of toxin synthesis. On the other hand, acidic culture conditions below pH 3.7 (e.g., acidification of the medium by fungal catabolism) are prerequisites for activation of trichothecene production if defined media are used (Nakajima et al., 2020). Although the ingredients differ significantly among products, yeast extract is often used as a source of nitrogen and micronutrients for the preparation of complex media. When a good manufacturing lot of yeast extract product is used, trichothecene biosynthesis is induced more strongly than with the use of defined media, despite the culture pH remaining at higher conditions. Specifically, *F. graminearum* produces trichothecenes on liquid YS_60 medium (0.1% [w/v] yeast extract, 6% [w/v] sucrose) with a pH above 3.7 (Nakajima et al., 2014). However, a disadvantage is that the mycotoxin-inducing activity differs considerably among the brands and lots of the yeast extract products. Complex media prepared with certain yeast extract products cannot induce trichothecenes by *F. graminearum*, posing difficulty in their utilization for biosynthesis regulation studies (Sørensen and Sondergaard, 2014; Tanaka et al., 2019). Interestingly, extraction of the complex media with ethyl acetate, followed by removal of the solvent fraction, results in increased trichothecene accumulation. Hence, several ethyl acetate-extractable substances in yeast extract negatively affects trichothecene production (Tanaka et al., 2019).

Utilization of defined media provides an advantage over the complex media in that their ingredients are consistent with each preparation, which is important for the experimental reproducibility of results. However, the pH of defined media generally tends to change more drastically than that of complex media. A key challenge in the utilization of defined media optimized for trichothecene production (Gardiner et al., 2009a) is the extreme acidification of the culture associated with fungal growth, which could misrepresent the involvement of factors that determine the culture pH, such as the catabolism of polyamines, in trichothecene induction (Nakajima et al., 2020). Hence, carefully worked-out plans will be necessary to evaluate the role of chemical and biological factors, whose direct impacts on trichothecene regulation are much less significant than their indirect and non-specific effects *via* pH.

### Example 1: Polyamines are not inducers of trichothecene production in axenic culture

Polyamines are aliphatic amines that are involved in stress responses in plants (Alcázar et al., 2006). A previous study has reported that trichothecene production *in vitro* was significantly lower compared to *in planta*, which led to the hypothesis that there are signals from host plants, such as polyamines, that act as inducers of trichothecene biosynthesis (Gardiner et al., 2009a). During the culture of *F. graminearum* under different amino acids and amines as the sole nitrogen sources, agmatine and putrescine drastically activated *Tri5* expression and trichothecene production, which motivated Gardiner and co-workers to speculate that such polyamines from plant polyamine biosynthetic pathways act as *in planta* signals for induction of trichothecene biosynthesis (Gardiner et al., 2009a).

However, when agmatine and putrescine were added in micromolar concentrations to *F. graminearum* JCM 9873 (15-ADON chemotype) on liquid YG_60 medium (0.1% [w/v] yeast extract, 6% [w/v] glucose), trichothecene biosynthesis was not induced (Nakajima et al., 2020). In contrast, other known trichothecene biosynthesis activators, such as sucrose (Nakajima et al., 2016) and NPD12671 chemical compound (Maeda et al., 2018), were effective in induction at micromolar concentrations in the same complex medium, demonstrating that such polyamines are not inducers (Nakajima et al., 2020). Additionally, in media containing either agmatine or l-Gln as the sole nitrogen source, 15-ADON production levels were similar between these two nitrogen sources when the pH of the l-Gln culture was frequently adjusted to the pH of the agmatine culture (Nakajima et al., 2020). Furthermore, the agmatine culture could not induce trichothecene biosynthesis when liquid cultures were maintained at a pH above 4 (Nakajima et al., 2020). Consequently, the natural pH decrease in the agmatine culture appears to be the factor responsible for inducing trichothecene biosynthesis. Together with the fact that the polyamines agmatine and putrescine are unable to induce trichothecene production at micromolar amounts, polyamines are not suitable to be defined as inducers unless their inducing abilities are proven under carefully controlled and considered culture conditions.

### Example 2: *FgAreA*, a nitrogen regulatory gene, is not essential for trichothecene biosynthesis

AreA is a GATA family transcription factor involved in the regulation of the utilization of secondary nitrogen sources (Tudzynski et al., 1999), and the ortholog *FgAreA* was reported to be indispensable for the utilization of most amino acids by *F. graminearum* strain PH-1(Giese et al., 2013). To study the effect of *FgAreA* deletion on trichothecene biosynthesis, wild-type (JCM 9873) and Δ*FgareA* mutant strains were cultured on liquid l-Gln medium with gyratory shaking. In accordance with previous reports on the role of FgAreA in secondary metabolism in strains PH-1 (Giese et al., 2013) and GZ3639 (Min et al., 2012), trichothecene was not detected in Δ*FgareA* cultures (Nakajima et al., 2020). Thus, FgAreA appears to be essential for trichothecene production. However, the time-dependent pH profiles of the liquid cultures were considerably different between the strains due to variations in growth rates, and the pH profiles of the Δ*FgareA* culture could not be superimposed on that of the wild-type by increasing the inoculum size (Nakajima et al., 2020). In fact, the pH of the Δ*FgareA* culture never fell below pH 3.7, a condition which we observe to be necessary to trigger trichothecene biosynthesis in defined media. When the experiment was performed with an l-Gln liquid medium constantly maintained at pH 2.5, trichothecene production by the Δ*FgareA* strain was comparable to that of the wild-type (Nakajima et al., 2020). The results showed that FgAreA is dispensable for trichothecene production and that the contribution of FgAreA to biosynthesis regulation depends on the pH of the cell culture medium. Since low pH affects trichothecene biosynthesis to a greater extent than FgAreA, when studying the roles of FgAreA, pH profiles must be considered during the experimental design and interpretation of results.

### Example 3: Overexpression experiment of the *WOR1* ortholog *Fgp1* under certain culture conditions could mislead its role in trichothecene biosynthesis

*WOR1* is a master transcriptional regulator of the white-opaque switching in *Candida albicans* (Zhang et al., 2014), and its homologs in various plant pathogenic fungi play pleiotropic roles, such as regulation of pathogenicity, conidiation, secondary metabolism, and other biological processes (Michielse et al., 2009, 2011; Jonkers et al., 2012; Santhanam and Thomma, 2013; Chen et al., 2014; Okmen et al., 2014). The *WOR1* ortholog in *F. graminearum* PH-1, *Fgp1*, was demonstrated to be essential for pathogenicity and trichothecene biosynthesis; revealing it plays a regulatory role in the expression of *Tri* genes (Jonkers et al., 2012). There is a conserved threonine-67 (T67) residue within the putative protein kinase A (Pka) phosphorylation site of Fgp1. As mutants containing a T67A mutation exhibit dramatically impaired virulence similar to the deletion mutant, phosphorylation of this residue is suggested to be important in the activation of Fgp1 (Yu et al., 2015). To further analyze and elucidate the role of *Fgp1*, we constructed an *Fgp1* overexpressor (Δ*Fgp1* + P*_GPD_::Fgp1*; *Fgp1* connected to a glyceraldehyde 3-phosphate dehydrogenase [*GPD*] promoter) strain using an *Fgp1* deletion (Δ*Fgp1*) host strain derived from JCM 9873, in addition to the complemented (Δ*Fgp1 + Fgp1*) and phosphorylation site mutant (Δ*Fgp1 + Fgp1*^T67A^) strains (Supplementary Figure 1).

**Figure 1:**
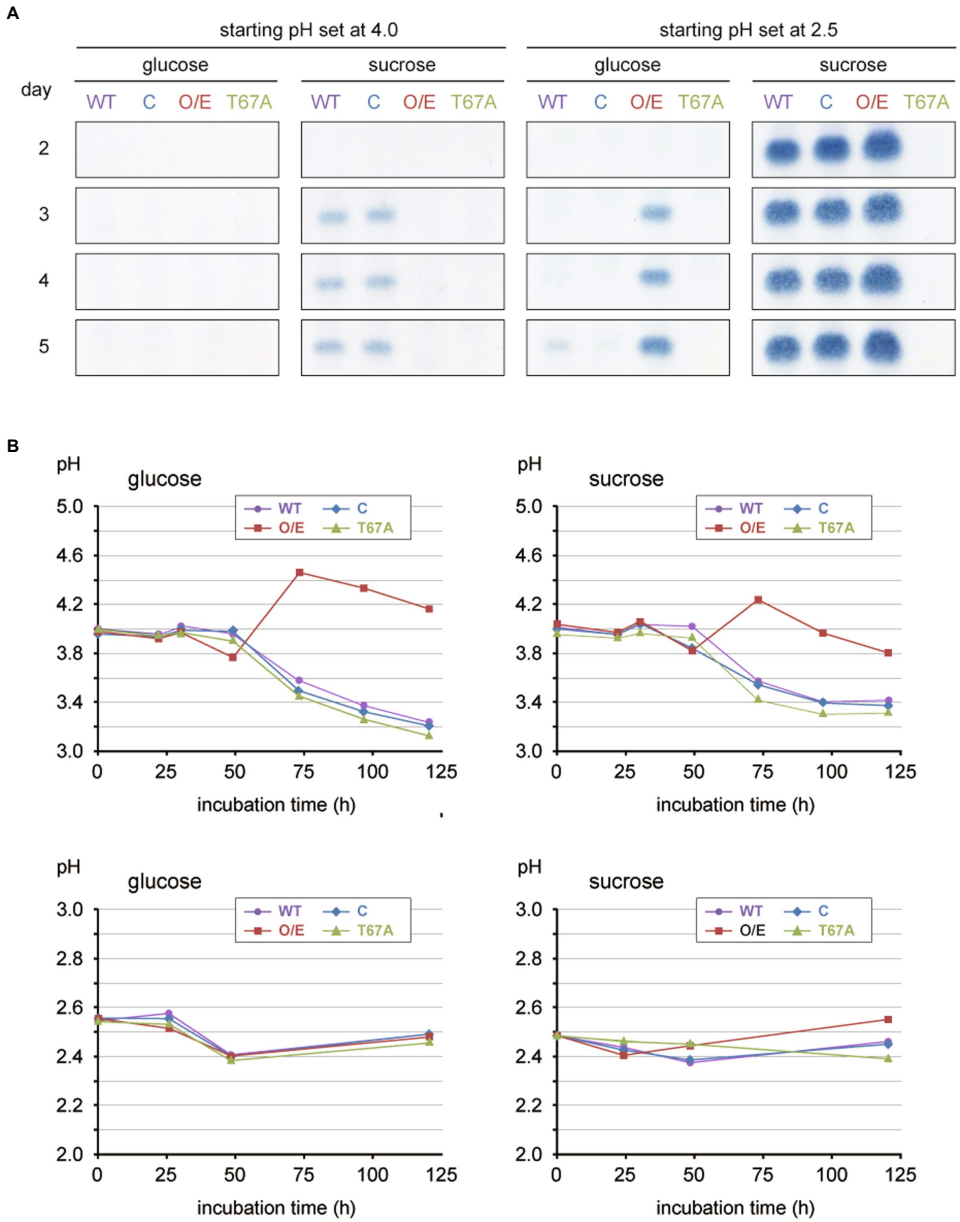
Toxin production assay of the various *Fgp1* mutant strains with two independent parameters, carbon source (glucose and sucrose) and initial pHs (4 and 2.5), cultured on 30 mL of medium in a 100-mL Erlenmeyer flask with gyratory shaking (135 rpm) at 25°C. **(A)** Each of the four strains tested (wild-type, WT; complemented, C; overexpressor, O/E; phosphorylation site mutant, T67A) was cultured on the defined media with amino acids mixture nitrogen source (Supplementary Table 1), and the carbon source and initial pH as indicated at the top. Each culture was sampled on days 2, 3, 4 and 5 of incubation. TLC panels show the spots of 15-ADON extracted from 500 μL of the medium with ethyl acetate. **(B)** Time course of pH changes of each fungal culture. When cultured at initial pH of 4, the wild-type, complemented, and T67A strains exhibited similar pH profiles, while the overexpressor displayed an increase in pH and deviated from the other three strains (upper graphs). When cultured at initial pH of 2.5, all four strains exhibited similar pH profiles (lower graphs).

When cultured axenically on liquid defined media with 3% (w/v) glucose and at a starting pH of 4 (see Supplementary Table 1 for medium composition), none of the strains produced 15-ADON (Figure 1A; panels on the first column). In the presence of sucrose in exchange for glucose, the wild-type and complemented strains produced 15-ADON, whereas the overexpressor did not (Figure 1A; panels on the second column). Interestingly, the pH profile of the overexpressor remarkably deviated from those of the other strains (Figure 1B; upper graphs); the *Fgp1* overexpressor showed a higher pH (above 4.2) as the growth reached the stationary phase and the pH never decreased below 3.7 over the entire growth phase. As *WOR1* homologs play pleiotropic roles, the large amount of Fgp1 present could result in metabolism that differed greatly from that of the wild-type on this particular defined medium and subsequent deviation in the pH profile of the culture. Since the pH of the culture medium has a significant impact on trichothecene biosynthesis in *F. graminearum* (Gardiner et al., 2009b), it is essential to observe trichothecene biosynthesis in all tested strains under the same pH condition. Thus, all four strains were then tested in media at a pH of 2.5 instead, where the pH profiles of all four strains were almost identical without any adjustment (Figure 1B; lower graphs). Under gyratory shaking on glucose medium, production of 15-ADON was marginal in the wild-type and complemented strains, while the overexpressor exhibited a marked increase in 15-ADON production (Figure 1A; panels on the third column). In the sucrose media, the wild-type, complemented, and overexpressor strains all exhibited high 15-ADON production levels (Figure 1A; panels on the fourth column), which was expected of due to the highly inducing conditions from the combination of low pH and presence of the inducer molecule sucrose. In agreement with the previous report on the functional importance of the T67 residue (Yu et al., 2015), no 15-ADON production by the T67A mutant was observed under all four culture conditions (Figure 1A). To confirm that the increased production of 15-ADON by the overexpressor is not limited to the extremely acidic culture conditions, the fungal strains were grown in YS_60 medium with higher culture pH. The results showed that the *Fgp1* overexpressor produced a greater amount of 15-ADON when the pH profile was similar to that of the wild-type (Supplementary Figure 2). Thus, we speculate that Fgp1 may play a role in leading to an active euchromatin structure around the trichothecene gene cluster, which subsequently triggers transcription of the cluster *Tri* genes.

If the effect of overexpressing *Fgp1* was interpreted based solely on the amount of the trichothecene produced, a conclusion on the effect of high *Fgp1* expression and subsequently the role of Fgp1 could be erroneously made. Our results therefore showed the importance of choosing appropriate experimental conditions to keep other factors that have a significant impact on trichothecene biosynthesis consistent, so that the effects of the factor studied can be observed more accurately.

### Example 4: Culturing of *F. graminearum* in a 24-well plate under gyratory shaking reveals the inducer role of sucrose in trichothecene biosynthesis

In liquid media, the size and shape of the culture vessel determine the morphological features of growing *F. graminearum* under gyratory shaking. Trichothecene biosynthesis is more strongly induced in flat mycelial flocs obtained by culturing in a 24-well plate than in dispersed mycelia obtained by culturing on the same medium in an Erlenmeyer flask (Nakajima et al., 2016). Notably, the trichothecene-inducing activity of oligosaccharides containing α-(1 → 2) (glucosyl/xylosyl)-fructosyl linkages, such as sucrose and kestose, could be observed only by culturing in 24-well plates (1 mL medium per well) (Nakajima et al., 2016). Although sucrose was previously believed to be required as a carbon source for the induction of trichothecene biosynthesis (Jiao et al., 2008; Kawakami et al., 2014), the use of a 24-well culture plate revealed that sucrose contributes even at lower concentrations of 100 μM. Consequently, this concentration is sufficiently low to be regarded as a specific inducer, rather than as a carbon source (Nakajima et al., 2016). Interestingly, if complex media are prepared by using a good yeast extract lot with the elimination of the ethyl acetate-extractable fraction, sucrose is unnecessary for trichothecene production, and *F. graminearum* cultured on liquid YF_60 medium (0.1% [w/v] yeast extract, 6% [w/v] fructose) accumulates a considerable amount of trichothecenes under gyratory shaking in a 24-well plate (Tanaka et al., 2019). Such a strong trichothecene-inducing culture condition is not appropriate for studying the inducer role of sucrose. Therefore, it is important to establish appropriate culture systems and conditions so that the specific role of sucrose in trichothecene biosynthesis can be observed.

## Mutational analyses of *Tri* genes in the trichothecene gene cluster core region

To date, the functional characterization of the *Tri* genes has mostly been investigated by conventional gene disruption techniques using vectors carrying the cloned homologous regions and a positive selection marker gene cassette. Both single and double crossover homologous recombination methods have been applied to knockout the *Tri* genes.

When the middle portion of a *Tri* gene is cloned into the vector and used for *Fusarium* transformation, two dysfunctional gene copies truncated at the 5′ and 3′ ends arise *via* single crossover homologous integration (method 1). Consequently, the distance between the upstream and downstream *Tri* genes is extended by the size of the gene disruption vector. The disruption vector can also be designed by inserting a marker cassette between the 5′ and 3′ regions of the *Tri* gene to be disrupted (or deleted if the homologous sequences do not include the gene-coding regions). The disruption vector backbone is eliminated *via* double crossover homologous integration, and the target *Tri* gene is replaced with the marker cassette (method 2). Thus, the length of the intergenic region in the trichothecene gene cluster may be changed. As discussed below, the extension of the cluster core region (refer to the Introduction section for the definition of the core region) remarkably affects *Tri* gene expression, presumably through changes in chromatin structure.

### Deregulation of trichothecene biosynthesis by structural changes of the cluster core region

**Figure 2:**
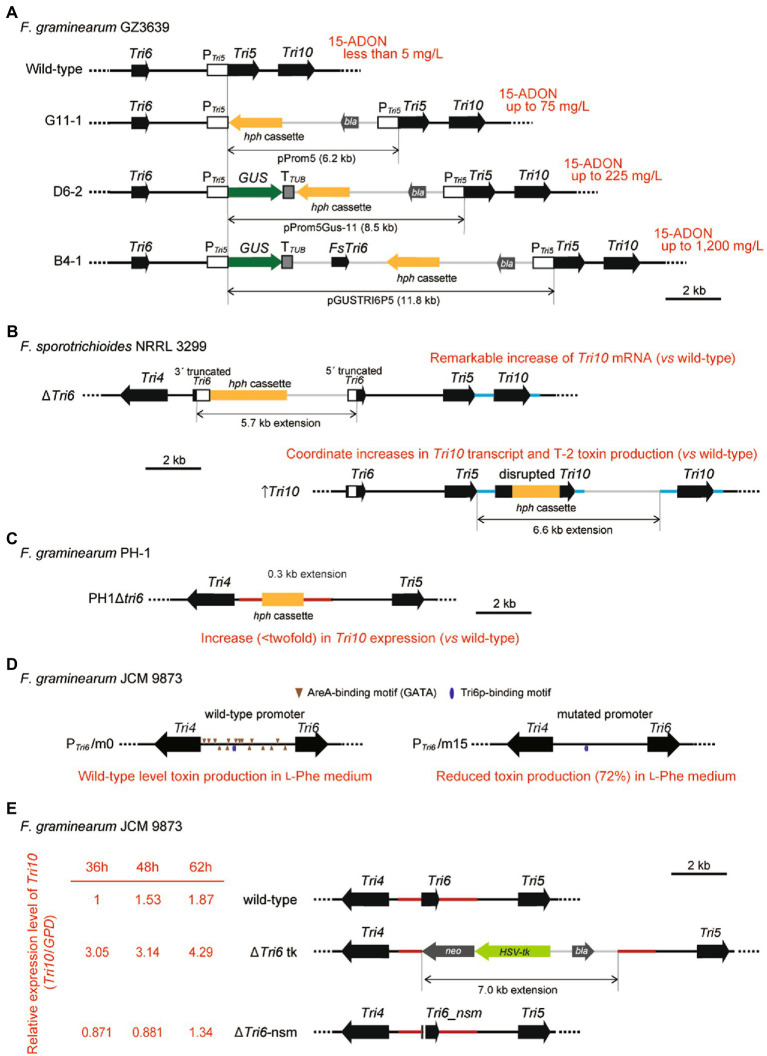
Genetic manipulations of the *Tri4*–*Tri6*–*Tri5*–*Tri10* locus in *Fusarium*. Deregulation of *Tri6* and *Tri10* occurs in cases when the targeted gene disruptions based on homologous recombination change the length of the core region of the trichothecene gene cluster. Vector integrations *via* single or double crossover homologous recombination for each subfigure are described in detail in Supplementary Figure 3. **(A)** Genetically engineered cluster core region of 15-ADON chemotype *F. graminearum* GZ3639 (Chen et al., 2000). The structure of the cluster core region is illustrated. Transformants, G11-1, D6-2, and B4-1, were obtained by homologous integration of vectors pProm5 (containing P*_Tri5_* alone), pProm5GUS-11 (containing P*_Tri5_* fused to *GUS*; P*_Tri5_::GUS*), and pGUSTRI6P5 (containing both P*_Tri5_::GUS* and *FsTri6*), respectively. Integration of pProm5, pProm5GUS-11, and pGUSTRI6P5 through the *Tri5* promoter region extends the *Tri5*–*Tri6* intergenic region by 6.2 kb (G11-1), 8.5 kb (D6-2), and 11.8 kb (B4-1), respectively. While the wild-type GZ3639 accumulates less than 5 mg/L of trichothecenes by shake culture on liquid medium with glucose, elongation of the cluster core region resulted in increased production of 15-ADON (up to 75 mg/L by G11-1, up to 225 mg/L by D6-2, and up to 1,200 mg/L by B4-1), irrespective of the presence or absence of *FsTri6* on the vector. Ectopic integration of pGUSTRI6P5 (e.g., up to 54 mg/l by strain B9-1; not shown in the subfigure) did not yield 15-ADON above the amount obtained by strain G11-1 lacking *FsTri6*. Notably, compared with strain B9-1, much stronger GUS activity was detected from strain B4-1 (50- to 100-fold increase), in which P*_Tri5_::GUS* is located at the cluster core region. **(B)** Genetically engineered cluster core region of *F. sporotrichioides* strain NRRL 3299 (Tag et al., 2001). The structure of the cluster core region of each derived mutant strain is illustrated. The resulting Δ*Tri6* strain contains a 3′-truncated *Tri6* gene followed by an *hph* cassette and then a 5′-truncated *Tri6* gene, and is expected to have a 5.7 kb extension at the *Tri6* region. The ↑*Tri10* and Δ*Tri10* strains were constructed from the same plasmid pTri10-1 which could undergo single or double crossover (Continued)FIGURE 2 (Continued)homologous recombination events at the upstream and/or downstream regions, respectively. The ↑*Tri10* strain contains a disrupted copy of *Tri10* followed by a functional copy of *Tri10* downstream of *Tri5* with a 6.6 kb extension, while the Δ*Tri10* strain contains only a disrupted copy of *Tri10* with a 1.6 kb extension in the same region (a Δ*Tri10* strain described in Supplementary Figure 3B only). **(C)** Disrupted *Tri6* in *F. graminearum* strain NRRL 31084 (PH-1) (Seong et al., 2009). The PH1*Δtri6* strain was generated by replacing the *Tri6* region with an *hph* cassette, resulting in a slight perturbation within the cluster core region. The region is extended by 0.3 kb in PH1*Δtri6*. **(D)** Wild-type and mutated *Tri6* promoters without structural perturbation of the cluster core region in *F. graminearum* strain JCM 9873 (Nakajima et al., 2020). P*_Tri6_*/m0 (complemented) and P*_Tri6_*/m15 (mutated) strains were designed by applying a two-step transformation process involving double crossover homologous recombination events. All fifteen 5′-GATA-3′ AreA-binding consensus sequences were mutated to 5′-GGTG-3′ in the wild-type *Tri4*–*Tri6* bidirectional promoter in P*_Tri6_*/m15. Both P*_Tri6_*/m0 and P*_Tri6_*/m15 strains are marker free and introduce no perturbation within the core cluster region. **(E)** Two types of *Tri6* disrupted mutants of *F. graminearum* strain JCM 9873. Both Δ*Tri6* tk (Nakajima et al., 2014) and Δ*Tri6*-nsm strains were generated to study the effect of *Tri6* deletion. The Δ*Tri6* tk strain was generated using a one-step transformation process and contains a selection marker cassette replacing the *Tri6* coding region, which results in a 7.0 kb extension in the cluster. The Δ*Tri6*-nsm strain was generated using a two-step transformation process and contains a dysfunctional copy of the *Tri6* gene containing a nonsense mutation, which does not result in perturbation within the cluster region. The relative expression level of *Tri10* (represented as *Tri10*/*GPD*) was determined using the expression of *GPD* as an endogenous reference in the same RNA samples. The *Tri10*/*GPD* ratio only increased in Δ*Tri6* tk, while that in Δ*Tri6*-nsm was similar to the expression level in the wild-type.

While *F. sporotrichioides* produces a considerable amount of trichothecene on a liquid shake culture with glucose as the carbon source, *F. graminearum* produces a limited amount of trichothecene under the same culture condition (Nakajima et al., 2016). In an attempt to boost trichothecene production, Chen et al. (2000) transformed *F. graminearum* with a series of vectors containing a promoter of *F. graminearum Tri5* (P*_Tri5_*), a β-d-glucuronidase (GUS) reporter gene, and/or a transcription unit of *F. sporotrichioides Tri6* (*FsTri6*) (Supplementary Figure 3A). The result revealed that trichothecene production is increased in either transformant, in which the *Tri5*–*Tri6* intergenic region was extended *via* homologous integration of the vector through the P*_Tri5_* sequence (Figure 2A). This study represents the first finding of a disordered regulation of trichothecene biosynthesis by changing the length of the cluster core region. Therefore, molecular genetic studies on the regulatory mechanisms of *Tri* gene expression should be performed without perturbating the core region of the gene cluster.

Similarly, in molecular genetic studies of *F. sporotrichioides*, Beremand’s group reported a deregulated expression of *Tri* genes in the cluster core region. When a 6.6 kb vector pTri10-1 was integrated into the *Tri5*–*Tri10* region *via* single crossover homologous recombination (Supplementary Figure 3B), the resulting transformants showed coordinate increases in *Tri10* transcript and T-2 toxin production over the wild-type levels (Figure 2B; ↑*Tri10*) (Tag et al., 2001). In addition, a remarkable increase of *Tri10* mRNA was observed on the northern blot of the *Tri6* disruption mutant (Figure 2B; strain Δ*Tri6* created by method 1 (Proctor et al., 1995)), in which the cluster core region was extended by 5.7 kb *via* single crossover homologous integration of the pTX6T-1 disruption vector at the *Tri6* locus (Supplementary Figure 3B). This observation led them to assume that transcription of *Tri10* is negatively regulated by Tri6p. Similarly, Kistler and co-workers reported an increase (<twofold) in *Tri10* expression (Figure 2C; PH1*Δtri6*) in their *Tri6* disruption mutant of *F. graminearum* (Supplementary Figure 3C; Seong et al., 2009), although smaller than that observed by Beremand’s group. The PH1*Δtri6* mutant created by double crossover homologous recombination (method 2) contains a *Tri5*–*Tri10* region elongated by 0.3 kb, which is much shorter than that of the *F. sporotrichioides* Δ*Tri6* mutant. Thus, the degree of extension of the cluster core region appears to affect the increase in *Tri10* expression level. Although the presence of Tri6p-binding motifs in the *Tri10* coding region (Supplementary Figure 6) led the researchers to hypothesize that they could be utilized to effectively block *Tri10* expression (Tag et al., 2001), the observed *Tri10* overexpression phenotype of the *Tri6* disruption mutants appears to be caused mainly by elongation of the *Tri5*–*Tri10* region, rather than *Tri6* inactivation (see next section).

### Introducing mutations into *Tri* genes at their native locus

Based on the issues described above, the structural organization of the trichothecene gene cluster should not be altered during the mutational analysis of the *Tri4*–*Tri6*–*Tri5*–*Tri10* region. To maintain the location and size of the genetic elements that reside in the cluster core region, mutations should be introduced into the native locus where the target genes are originally located at. Consequently, we use a two-step transformation process involving double crossover homologous recombination (Khang et al., 2005); in which self-cloning strains are generated by replacing the wild-type genetic elements with mutated alleles. In this process, marker-free mutants are obtained by positive selection and subsequent conditional negative selection against the *hph::tk* cassette, which contains a hygromycin B phosphotransferase gene (*hph*) that is translationally fused to a herpes simplex virus thymidine kinase gene (*tk*) (Maeda and Ohsato, 2017). This self-cloning strategy enabled the correct evaluation of the effect of mutations introduced at the native locus on the regulation of trichothecene biosynthesis. Using this strategy, our group mutated all fifteen 5′-GATA-3′ AreA-binding consensus sequences to 5′-GGTG-3′ in the wild-type *Tri4*–*Tri6* bidirectional promoter (Supplementary Figure 3D), and assessed precisely the degree of contribution of catabolizing l-Phe used as the sole nitrogen source of culture medium on trichothecene biosynthesis (Figure 2D; Nakajima et al., 2020). When generating knock-out strains, this strategy is also useful as the native gene can be replaced with a gene containing a nonsense mutation, instead of conventionally replacing the target gene with a selection marker cassette that is often of different lengths.

Given the altered trichothecene production caused by structural perturbation of the gene cluster core region (Chen et al., 2000), we question previous models on the roles of *Tri6* and *Tri10* in the transcriptional activation of *Tri* genes. As mentioned previously, several studies have reported that *Tri10* expression was elevated in *Tri6* deletion strains, thus concluding that *Tri10* was negatively regulated by *Tri6* (Tag et al., 2001; Seong et al., 2009). However, our pilot study comparing the *Tri10* expression between two types of *Tri6* disruptant strains, *Tri6* replaced with either a selection cassette (strain Δ*Tri6* tk) or *Tri6_nsm* containing a nonsense mutation (strain Δ*Tri6*-nsm) (Supplementary Figure 3E), revealed that *Tri10* expression was elevated only in the Δ*Tri6* tk strain, whereas that in Δ*Tri6*-nsm was similar to that in the wild-type (Figure 2E; see fold change of *Tri10*/*GPD*). This shows that the increase in *Tri10* expression in *Tri6* deletion mutants reported in previous studies (Tag et al., 2001; Seong et al., 2009) was likely due to the structural extension in the core gene cluster upstream of *Tri10* (Figures 2B,C), and the model which suggests that *Tri6* negatively regulates *Tri10* requires revision. Another group suggested that Tri10p and FgAreAp interact with each other and this interaction is possibly important for the activation of other *Tri* genes (Hou et al., 2015). To further examine whether *FgAreA* and *Tri10* deletion would have the same extent of effect due to the loss of such interaction, we investigated 15-ADON production in these mutant strains. At low pH of 2.5, the Δ*FgareA* strain (Nakajima et al., 2020) produced a considerable amount of 15-ADON, while the Δ*Tri10*-nsm strain, carrying *Tri10_nsm* with a nonsense mutation and without perturbing the gene cluster core region, produced no 15-ADON (Supplementary Figure 7). The result suggests that *Tri10* is much more important than *FgAreA* in the regulation of trichothecene biosynthesis and that the physical interaction between FgAreAp and Tri10p (Hou et al., 2015) is unessential.

Thus far, several studies have overlooked the impact of extension in the trichothecene gene cluster, thus, there is a need for revisions to improve our present knowledge of the regulatory mechanism of *Tri* gene expression. In addition, future studies must consider these effects. Genome editing using Clustered Regularly Interspaced Short Palindromic Repeats (CRISPR)-associated (Cas) nucleases may be a promising tool for genetic manipulation of *F. graminearum* without perturbations in the cluster core region, but in the absence of selection, the recovery of mutants is relatively infrequent and poses a problem for efficient research currently (Gardiner and Kazan, 2018). Finally, we close this perspective paper by presenting a novel idea that could lead to a deeper understanding of the complex regulation of *Tri6*.

## Future perspectives

Results from the studies performed by our group suggest that *Tri6* transcription is under two types of regulation as follows: Tri6p-independent initial activation and subsequent Tri6p-dependent feedback activation by itself (Maeda et al., 2018). For molecular genetic studies to discriminate between the two modes of regulation, we have constructed a strain that contains a copy of *Tri6_nsm* at the native locus and a copy of highly-expressing synonymous *Tri6* gene at an ectopic locus (Δ*Tri6*-nsm/syn*Tri6*) by using the Δ*Tri6*-nsm strain (described in the above subsection; see also Supplementary Figure 3E) as the parent. In the Δ*Tri6*-nsm strain, no Tri6p is produced; thus, Tri6p-dependent activation cannot occur. Further we established that nonsense-mediated *Tri6_nsm* mRNA decay (Wen and Brogna, 2010) does not occur in Δ*Tri6*-nsm, as suggested by the observation of similar levels of Tri6p-independent transcription of mutated *Tri6*, made from the native locus in mutant strains with dysfunctional zinc finger domains. A copy of the synonymous *Tri6* gene, which has a different DNA sequence from that of the native *Tri6* gene but encodes the same amino acid sequence, is highly expressed from an ectopic locus. By comparing the expression level of *Tri6_nsm* at the native locus in both the Δ*Tri6*-nsm and Δ*Tri6*-nsm/syn*Tri6* strains, the Tri6p-independent initial activation and subsequent Tri6p-dependent activation could be differentiated while studying the effects of the associated factors, thus, providing further clarity and insight into the regulatory mechanism of trichothecene biosynthesis.

## Data availability statement

The original contributions presented in the study are included in the article/Supplementary material, further inquiries can be directed to the corresponding author.

## Author contributions

MXXL, YN, and MK conceived the study. MXXL, YN, KM, and NK performed the wet experiments and analyzed data. MXXL and MK wrote and elaborated the manuscript. MXXL, YN, KM, and MK designed the figures. All authors read and approved the final manuscript.

## Funding

This work was supported by a grant from the JSPS KAKENHI, Grant-in-Aid for Scientific Research C (22 K05379), and a research grant from IFO, the Institute for Fermentation, Osaka (G-2021-2-098).

## Conflict of interest

The authors declare that the research was conducted in the absence of any commercial or financial relationships that could be construed as a potential conflict of interest.

## Publisher’s note

All claims expressed in this article are solely those of the authors and do not necessarily represent those of their affiliated organizations, or those of the publisher, the editors and the reviewers. Any product that may be evaluated in this article, or claim that may be made by its manufacturer, is not guaranteed or endorsed by the publisher.
