# Supplementary material for: Regulatory mechanism of trichothecene biosynthesis in Fusarium graminearum
Source: Front Microbiol. 2023 Apr 17;14:1148771. doi: 10.3389/fmicb.2023.1148771 (PMC10149712; doi:10.3389/fmicb.2023.1148771)
Supplement: Supplementary file 1 [file Data_Sheet_1.pdf]

Supplementary Material

Regulatory Mechanism of Trichothecene Biosynthesis in *Fusarium graminearum*

Maydelene Xiao Xuan Liew[1], Yuichi Nakajima[1], Kazuyuki Maeda[1], Naotsugu Kitamura[1], Makoto Kimura[1,*]

[1]Department of Applied Biosciences, Graduate School of Bioagricultural Sciences, Nagoya University, Furo-cho, Chikusa-ku, Nagoya, Aichi 464-8601, Japan

Correspondence

Makoto Kimura

mkimura@agr.nagoya-u.ac.jp

Supplementary Table 1 | Composition of defined medium used in the study of 15-ADON production in various *F. graminearum* transformant strains.

Defined medium for submerged culture

Element	Concentration
Carbon source	30 g/L
KH_2PO_4	1 g/L
KCl	0.5 g/L
$MgSO_4 \cdot 7H_2O$	0.5 g/L
Trace elements[1]	0.2 mL/L
$FeSO_4 \cdot 7H_2O$	10 mg/L
Nitrogen source [1,2]	5 mM

[1] filter-sterilized.

[2] amino acids mixture or L-Glutamine.

5000 × Trace elements

Element	Concentration
Citric acid	5 g/100 mL
$MnSO_4$	50 mg/100 mL
$ZnSO_4 \cdot 6H_2O$	5 g/100 mL
H_3BO_3	50 mg/100 mL
$Na_2MoO_4 \cdot 2H_2O$	50 mg/100 mL
$CuSO_4 \cdot 5H_2O$	250 mg/100 mL

Amino acids mixture

Element	Concentration
L-Glutamic acid	1 mM
L-Glutamine	1.5 mM
L-Leucine	2.5 mM

Supplementary Table 2 | List of primers used for qRT-PCR in the study of *Tri10* expression in various *F. graminearum* strains.

Primer name	Sequence (5'-3')	Description
JCM_Tri10_qRT-F	TGTCGCCTCATACGACCTC	Quantitative real-time PCR primers for the analysis of *Tri10* expression
JCM_Tri10_qRT-R	ATGACGGAACTCTTCAGGTCTT	
qRT-Gpd_Fw	CGAAGTTGTCGTTGAGGGAG	Quantitative real-time PCR primers for the analysis of *GPD* expression
qRT-Gpd_Rev	GACAACGAGTGGGGTTACTCC	

(A)

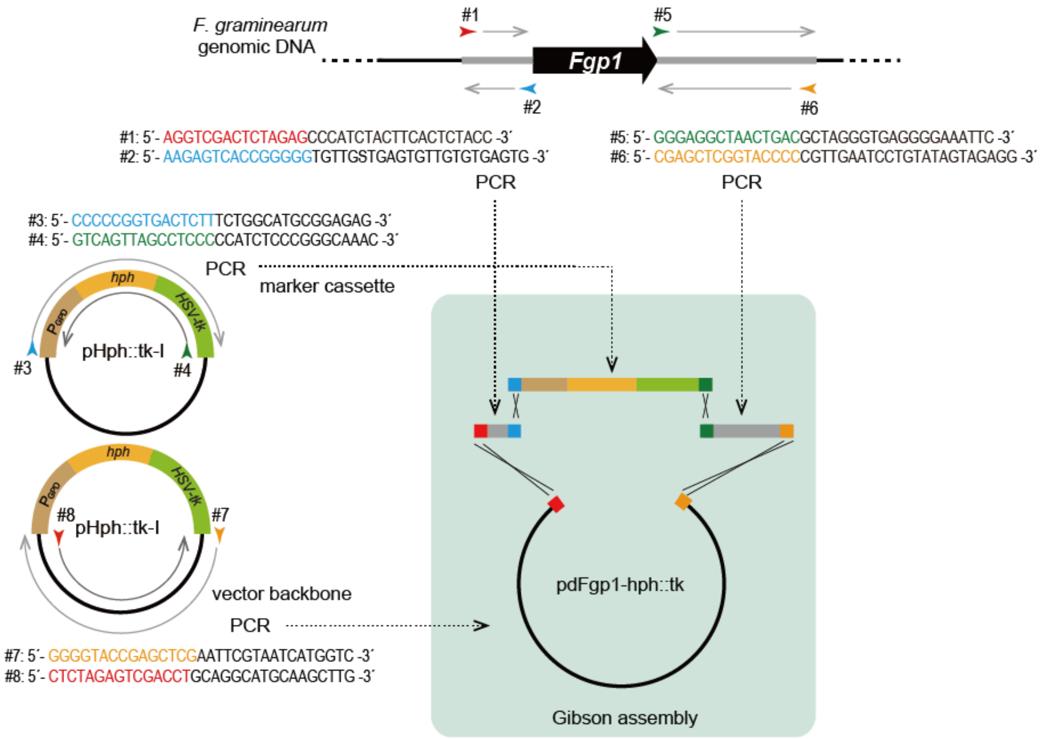
F. graminearum genomic DNA
#1
#5
Fgp1
#2
#6
#1: 5´- AGGTCGACTCTAGAGCCCATCTACTTCACTCTACC -3´
#2: 5´- AAGAGTCACCGGGGGTGTTGSTGAGTGTTGTGTGAGTG -3´
#5: 5´- GGGAGGCTAACTGACGCTAGGGTGAGGGGAAATTC -3´
#6: 5´- CGAGCTCGGTACCCCCGTTGAATCCTGTATAGTAGAGG -3´
PCR
PCR
#3: 5´- CCCCCGGTGACTCTTTCTGGCATGCGGAGAG -3´
#4: 5´- GTCAGTTAGCCTCCCCCATCTCCCGGGCAAAC -3´
PCR
marker cassette
hph
HSV-tk
pHph::tk-I
#3
#4
#8
#7
vector backbone
PCR
#7: 5´- GGGGTACCGAGCTCGAATTCGTAATCATGGTC -3´
#8: 5´- CTCTAGAGTCGACCTGCAGGCATGCAAGCTTG -3´
pdFgp1-hph::tk
Gibson assembly

(B)

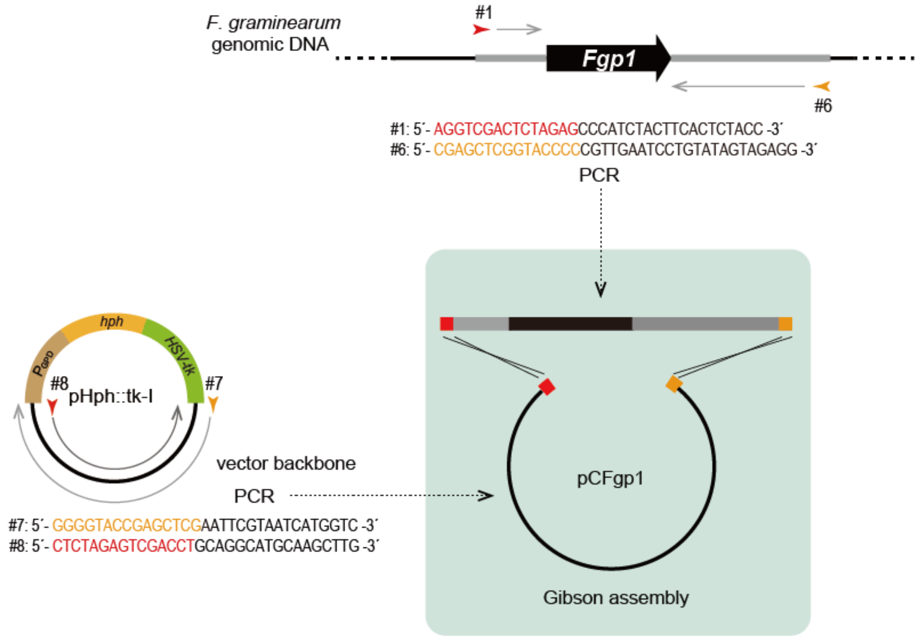
F. graminearum genomic DNA
#1
Fgp1
#6
#1: 5´- AGGTCGACTCTAGAGCCCATCTACTTCACTCTACC -3´
#6: 5´- CGAGCTCGGTACCCCCGTTGAATCCTGTATAGTAGAGG -3´
PCR
hph
HSV-tk
#8
pHph::tk-I
#7
vector backbone
PCR
#7: 5´- GGGGTACCGAGCTCGAATTCGTAATCATGGTC -3´
#8: 5´- CTCTAGAGTCGACCTGCAGGCATGCAAGCTTG -3´
pCFgp1
Gibson assembly

(C)

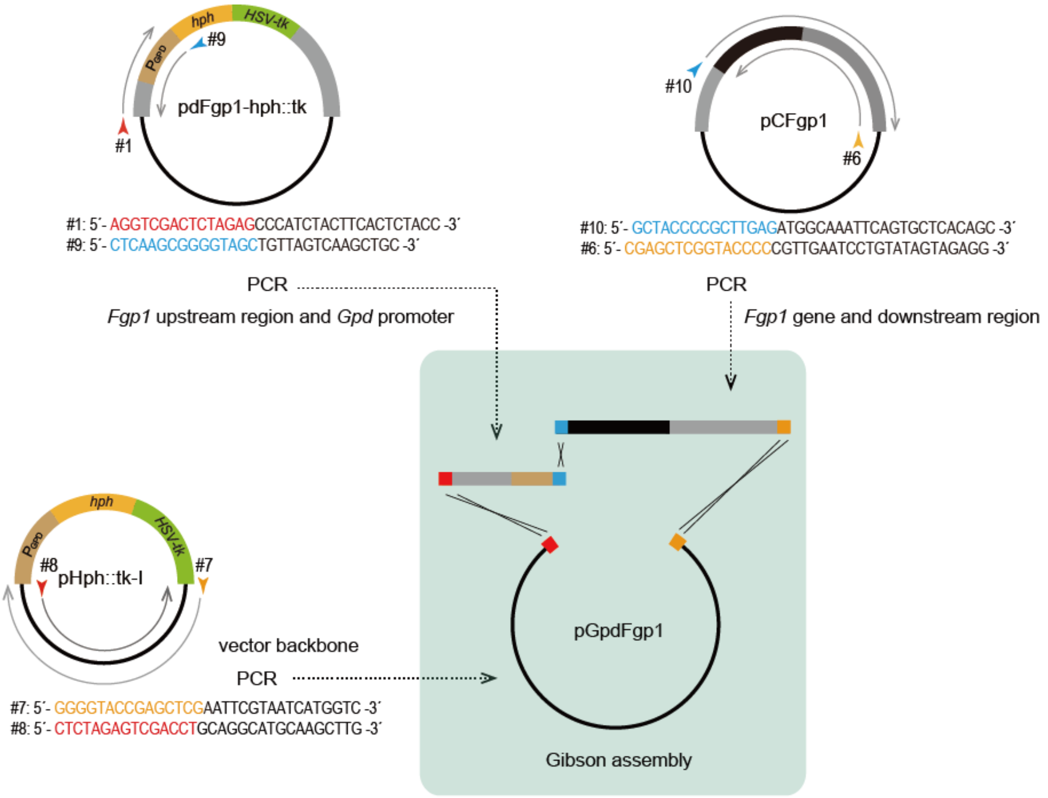
hph
HSV-tk
PGPD
#9
pdFgp1-hph::tk
#1
#10
pCFgp1
#6
#1: 5´- AGGTCGACTCTAGAGCCCATCTACTTCACTCTACC -3´
#9: 5´- CTCAAGCGGGGTAGCTGTTAGTCAAGCTGC -3´
#10: 5´- GCTACCCCGCTTGAGATGGCAAATTCAGTGCTCACAGC -3´
#6: 5´- CGAGCTCGGTACCCCCGTTGAATCCTGTATAGTAGAGG -3´
PCR
Fgp1 upstream region and Gpd promoter
PCR
Fgp1 gene and downstream region
#8
pHph::tk-I
#7
vector backbone
PCR
#7: 5´- GGGGTACCGAGCTCGAATTCGTAATCATGGTC -3´
#8: 5´- CTCTAGAGTCGACCTGCAGGCATGCAAGCTTG -3´
pGpdFgp1
Gibson assembly

(D)

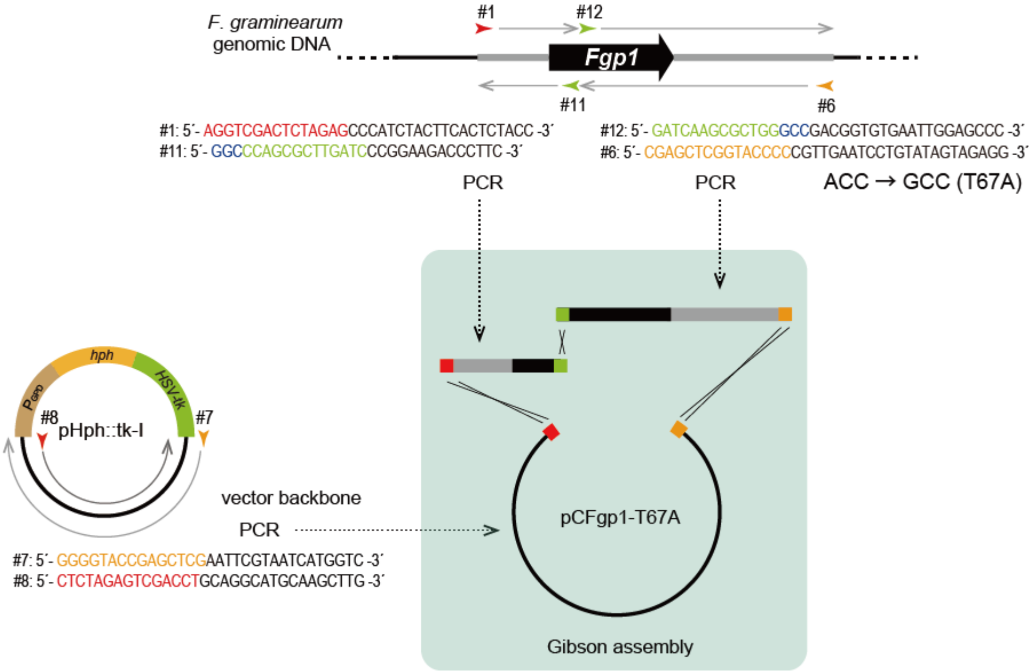
F. graminearum
genomic DNA
#1
#12
Fgp1
#11
#6
#1: 5´- AGGTCGACTCTAGAGCCCATCTACTTCACTCTACC -3´
#11: 5´- GGCCCAGCGCTTGATCCCGGAAGACCCTTC -3´
#12: 5´- GATCAAGCGCTGGGCCGACGGTGTGAATTGGAGCCC -3´
#6: 5´- CGAGCTCGGTACCCCCGTTGAATCCTGTATAGTAGAGG -3´
PCR
PCR
ACC → GCC (T67A)
hph
HSV-tk
PGPD
#8
pHph::tk-I
#7
vector backbone
PCR
#7: 5´- GGGGTACCGAGCTCGAATTCGTAATCATGGTC -3´
#8: 5´- CTCTAGAGTCGACCTGCAGGCATGCAAGCTTG -3´
pCFgp1-T67A
Gibson assembly

(E)

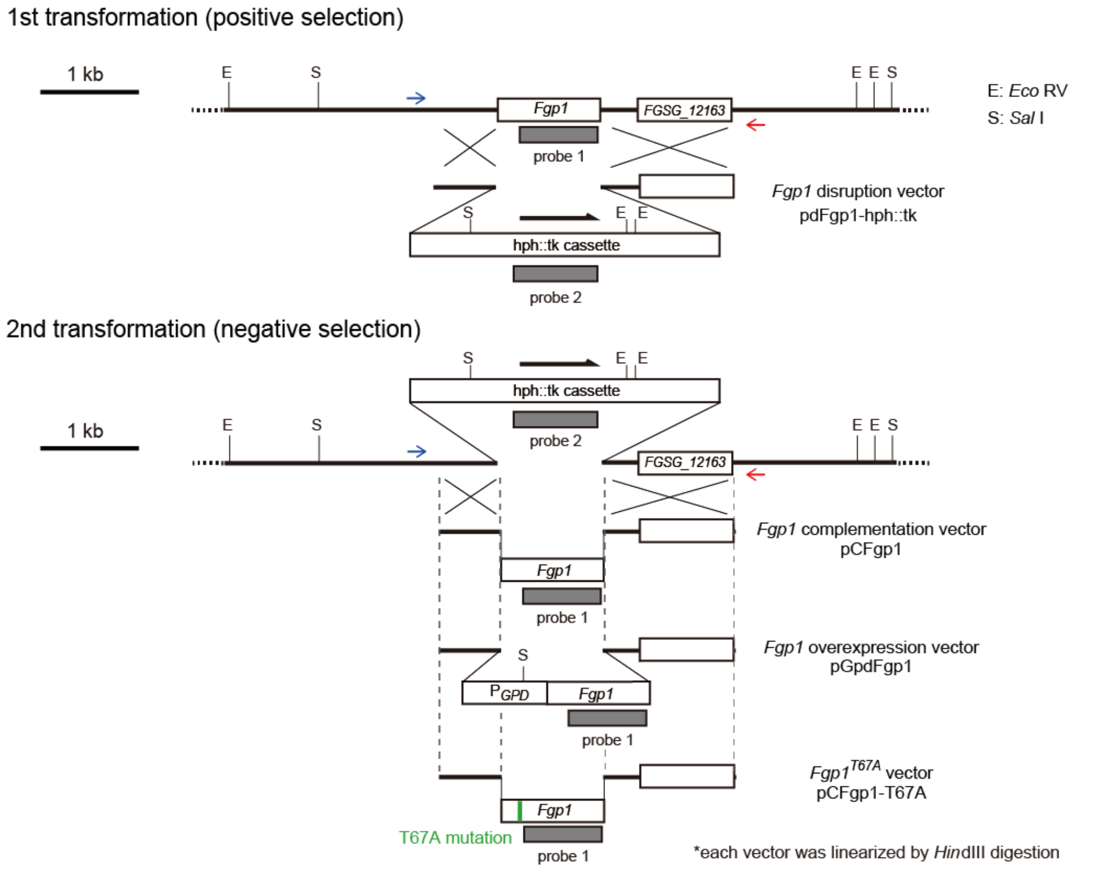

(F)

PCR

→ Fgp1-Long_S:
5'-GGCCTTTCTCAGCTTGACCTAGGGGTCTTC-3'

→ Fgp1-Long_AS:
5'-TCCTCGTCTTGTCTTTGTGTTGCTAGCCTG-3'

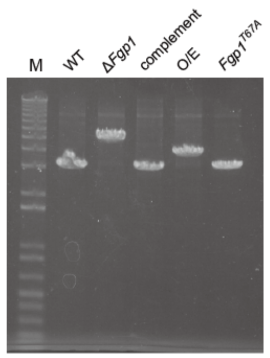

Correct amplicon size

WT (3,183 bp)
ΔFgp1 (5,165 bp)
com (3,183 bp)
O/E (4,045 bp)
Fgp1^{T67A} (3,183 bp)

Southern blot analysis

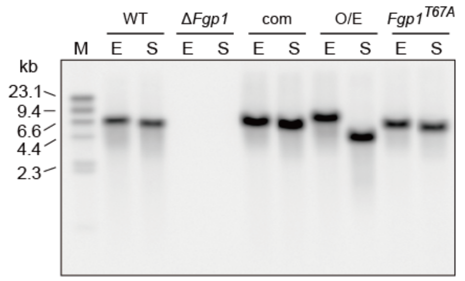

Predicted band sizes

WT (E: 6,253 bp, S: 5,720 bp)
ΔFgp1 (E: none, S: none)
com (E: 6,253 bp, S: 5,720 bp)
O/E (E: 7,115 bp, S: 4,282 bp)
Fgp1^{T67A} (E: 6,253 bp, S: 5,720 bp)

probe 1

Primers for probe 1 amplification
Fgp1-probe_F: 5'-GCCGAATCTTGGCAACTTC-3'
Fgp1-probe_R: 5'-GCCATTTTGGATGGCATGGG-3'

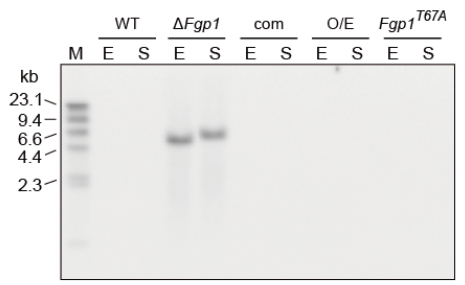

Predicted band sizes

WT (E: none, S: none)
ΔFgp1 (E: 4,799 bp, S: 5,399 bp)
com (E: none, S: none)
O/E (E: none, S: none)
Fgp1^{T67A} (E: none, S: none)

probe 2

Primers for probe 2 amplification
hph-PL1: 5'-AGCTGCGCCGATGGTTTCTACAA-3'
hph-PR1: 5'-GGGGCGTCGGTTTCCACTATCG-3'

Supplementary Figure 1 | Construction of homologous recombination vectors and generation of various *Fgp1* transformants. (A) Construction of pdFgp1-hph::tk. Four pairs of PCR primers (primers #1 – #8), with 15 bp overhangs necessary for Gibson Assembly, were designed as shown in the figure. Positive-negative selection marker cassette, containing a glyceraldehyde 3-phosphate dehydrogenase (*GPD*) promoter (from *Aspergillus nidulans* AN8041) fused to *hph::tk*, was amplified from pHph::tk-I (Maeda and Ohsato 2017) with primers #3 × #4. Vector backbone was amplified from the same plasmid with primers #7 × #8. Upstream and downstream regions of *Fgp1* were obtained using genomic DNA as template with primers #1 × #2 and #5 × #6, respectively. The four PCR fragments were connected by Gibson Assembly using the NEBuilder HiFi DNA Assembly Master Mix (New England BioLabs, Ipswich, USA). (B) Construction of pCFgp1. Two pairs of PCR primers (primers #1, #6 − #8), with 15 bp overhangs necessary for Gibson Assembly, were designed as shown in the figure. Vector backbone was amplified from pHph::tk-I with primers #7 × #8, and the region from the upstream of *Fgp1* to its downstream was amplified using genomic DNA as template with primers #1 × #6. The two PCR fragments were assembled by Gibson Assembly. (C) Construction of pGpdFgp1. Three pairs of PCR primers (primers #1, #6 – #10), with 15 bp overhangs necessary for Gibson Assembly, were designed as shown in the figure. Vector backbone was amplified from pHph::tk-I with primers #7 × #8, and the *Fgp1* upstream region and the *GPD* promoter was amplified from pdFgp1-hph::tk with primers #1 × #9. The *Fgp1* gene and its downstream region was amplified from pCFgp1 using primers #10 × #6, and the three PCR fragments were assembled by Gibson Assembly. (D) Construction of pCFgp1-T67A. Three pairs of PCR primers (primers #1, #6– #8, #11– #12), with 15 bp overhangs necessary for Gibson Assembly, were designed as shown in the figure. Primers #11 and #12 contain a single base pair change that would introduce a point mutation into *Fgp1* for the replacement of threonine with alanine at the putative phosphorylation site. Vector backbone was

amplified from pHph::tk-I with primers #7 × #8, the region from the upstream of *Fgp1* to the putative phosphorylation site was amplified from the genome with primers #1 × #11, and the remaining region of *Fgp1* and its downstream region was amplified from the genome with primers #12 × #6. The three fragments were then assembled by Gibson Assembly. **(**E) Schematic diagram of the genomic structure of the various *Fgp1* strains generated: deletion strain (Δ*Fgp1*), complemented strain (Δ*Fgp1* + *Fgp1*; com), overexpressor strain (Δ*Fgp1* + P_{GPD}::*Fgp1*; O/E) and phosphorylation site disruptant (Δ*Fgp1* + *Fgp1*^{T67A}; *Fgp1*^{T67A}). (F) PCR and Southern blot verification of the *Fgp1* mutant strains. Expected sizes of amplicons were obtained after PCR with primers (red and blue) located outside of the homologous region (left panel). The *Fgp1* sequence of *Fgp1*^{T67A} strain was confirmed by DNA sequencing. Southern blot of genomic DNA digested with *Eco*RV and *Sal*I was hybridized with a DIG-labeled probe 1 and probe 2, which was prepared using a PCR DIG Probe Synthesis Kit (Roche Diagnostics GmbH, Mannheim, Germany) and primers described in the figure. Predicted sizes of single bands were detected for the *Eco*RV and *Sal*I digested DNA (right panel).

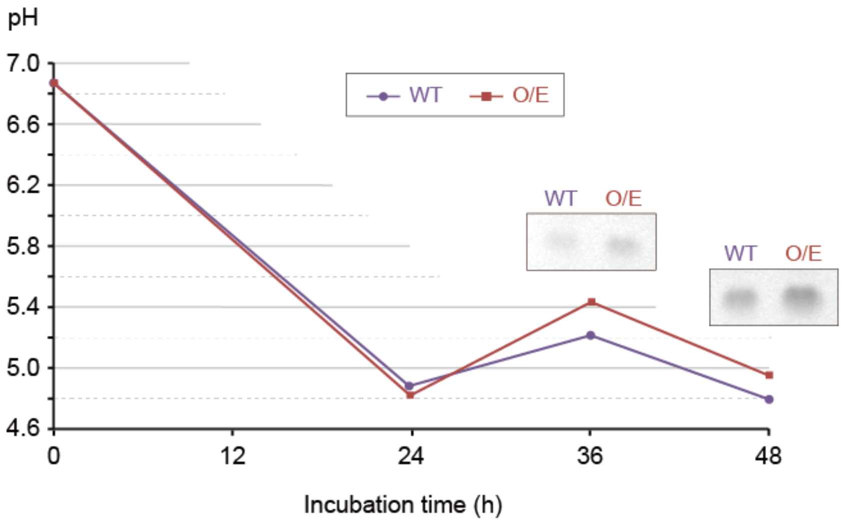

Supplementary Figure 2 | Toxin production assay of the wild-type (WT) and *Fgp1* overexpressor (O/E) strains, cultured in 30 mL of YS_60 medium (0.1% [w/v] yeast extract, 6% [w/v] sucrose) in a 100-mL Erlenmeyer flask with gyratory shaking (135 rpm) at 25ºC. Fresh conidia were inoculated into the YS_60 medium at a cell density of 1×10^4 conidia/mL. With this inoculum size and 24 h of incubation period, the mycelia are too premature to cause toxin accumulation. 15-ADON was extracted from 500 μL of each medium with ethyl acetate 36 and 48 hr after the inoculation. The insets represent TLC panels of 15-ADON that accumulated in fungal cultures, as detected by UV absorption at 254 nm. The pH profiles of each fungal culture was similar with each other and remained above 4.7, as shown in the graph.

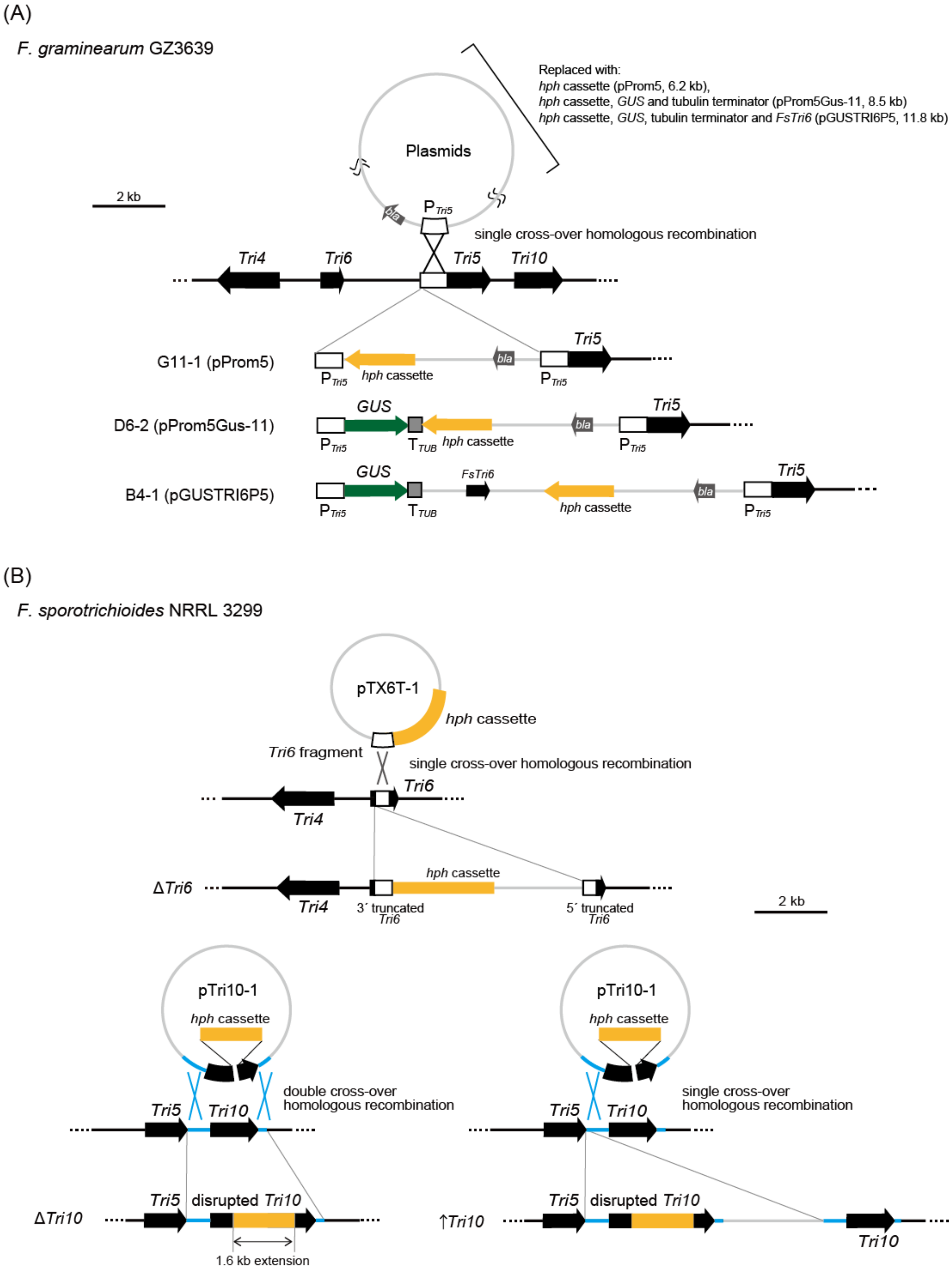
(A)
F. graminearum GZ3639
Replaced with:
hph cassette (pProm5, 6.2 kb),
hph cassette, GUS and tubulin terminator (pProm5Gus-11, 8.5 kb)
hph cassette, GUS, tubulin terminator and FsTri6 (pGUSTRI6P5, 11.8 kb)
Plasmids
2 kb
bla
P Tri5
single cross-over homologous recombination
Tri4
Tri6
Tri5
Tri10
G11-1 (pProm5)
hph cassette
D6-2 (pProm5Gus-11)
GUS
T TUB
B4-1 (pGUSTRI6P5)
FsTri6
(B)
F. sporotrichioides NRRL 3299
pTX6T-1
hph cassette
Tri6 fragment
ΔTri6
3´ truncated Tri6
5´ truncated Tri6
pTri10-1
double cross-over homologous recombination
single cross-over homologous recombination
ΔTri10
disrupted Tri10
1.6 kb extension
↑Tri10

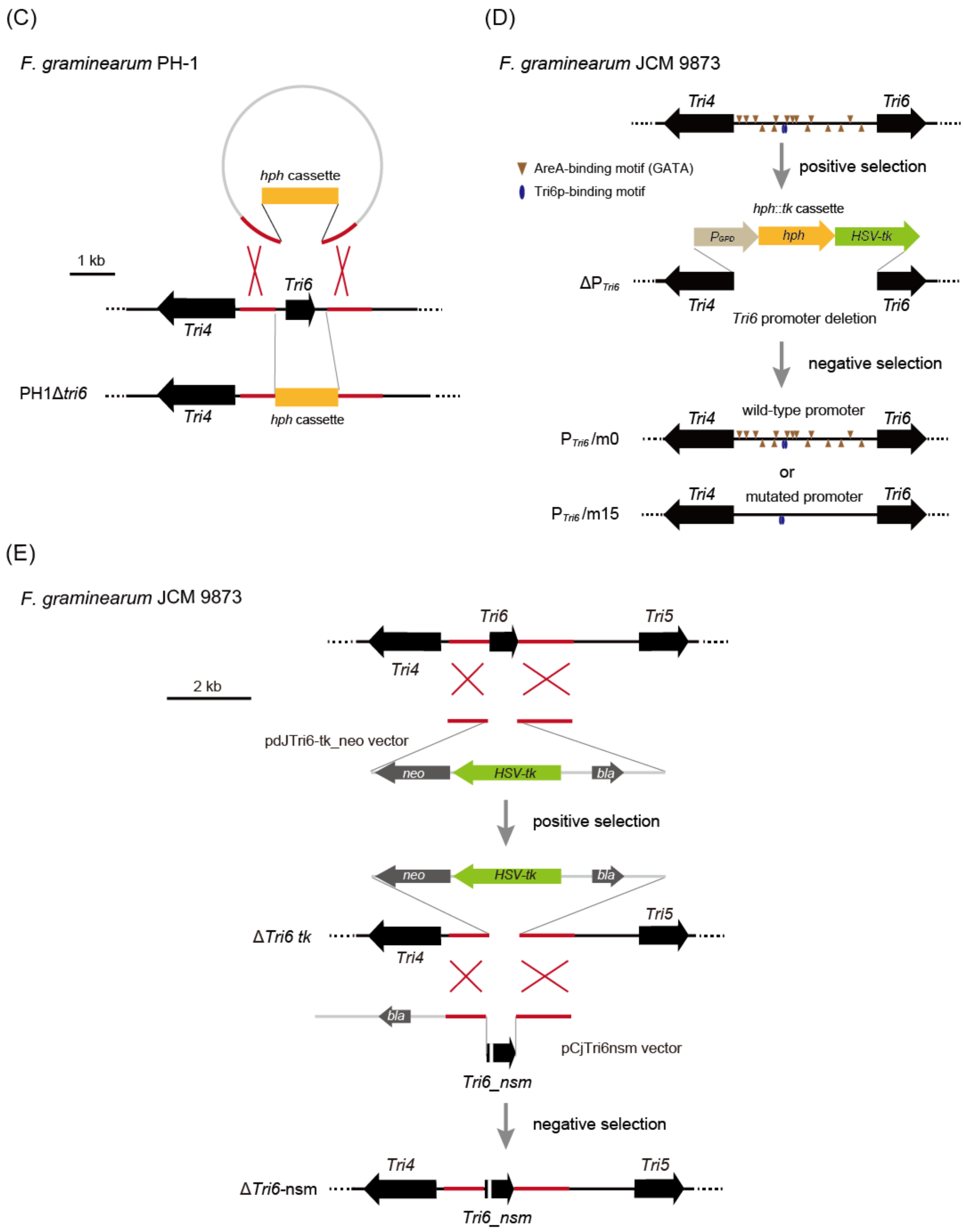

Supplementary Figure 3 | Illustration of genetic transformation of vectors via single or double crossover homologous recombination into the strains presented in Figure 2. (A) Manipulation of the trichothecene gene cluster of *F. graminearum* GZ3639 (Chen et al., 2000). The intergenic region between *Tri6* and *Tri5* is extended by the insertion of the

respective plasmids by a single crossover homologous recombination event. (B) Manipulation of the trichothecene gene cluster of *F. sporotrichioides* strain NRRL 3299 (Proctor et al., 1995; Tag et al., 2001). The core cluster region is extended when the respective plasmids are inserted via a single crossover (for plasmids pTX6T-1 and pTri10-1) or double crossover (for plasmid pTri10-1) homologous recombination events. (C) Manipulation of the trichothecene gene cluster in *F. graminearum* strain NRRL 31084 (PH-1) (Seong et al., 2009). The PH1Δ*tri6* strain was obtained via a double crossover homologous recombination event at upstream and downstream regions flanking *Tri6*, which led to a 0.3 kb extension of the core cluster region. (D) Manipulation of the *Tri6* promoter region in *F. graminearum* strain JCM 9873 (Nakajima et al., 2020). The strains were constructed using a two-step transformation process involving double crossover homologous recombination events, and are marker free with no perturbation within the core cluster region. (E) Manipulation of the trichothecene gene cluster in *F. graminearum* strain JCM 9873. The Δ*Tri6* tk strain was generated using a one-step transformation process (Nakajima et al., 2014), and contains a selection marker cassette replacing the *Tri6* coding region, which leads to 7.0 kb extension in the cluster. The Δ*Tri6*-nsm strain was generated using a two-step transformation process, and contains a dysfunctional copy of *Tri6* gene, which does not lead to perturbation in the cluster region. See **Supplementary Figure 4** for experimental details of the mutant strain construction and confirmation.

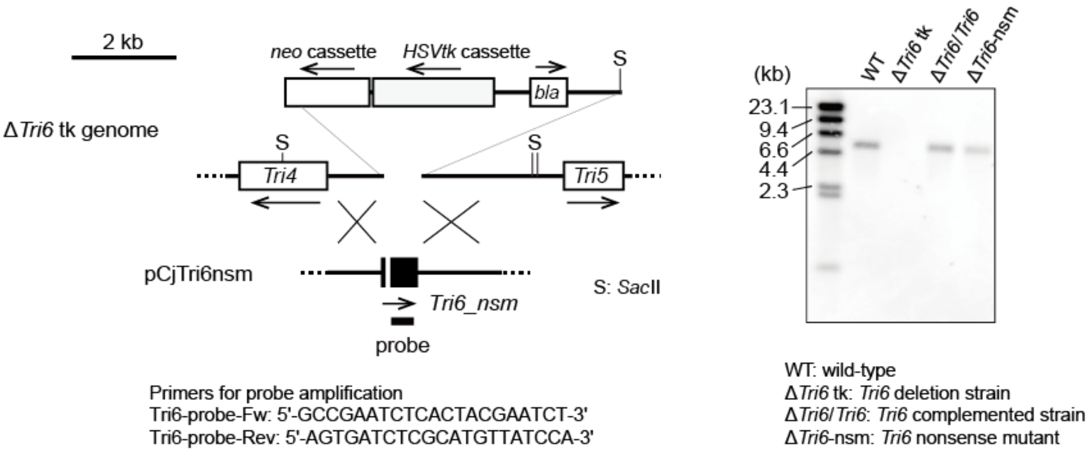

Supplementary Figure 4 | Generation of the Δ*Tri6*-nsm mutant strain. The *Tri6* disruption mutant, strain Δ*Tri6* tk (Nakajima et al., 2014), was transformed with pCjTri6nsm (**Supplementary Figure 5**) and a self-cloning strain carrying a mutated *Tri6* (*Tri6_nsm*) was screened by conditional negative selection with 2′-deoxy-5-fluorouridine (5-FdU) (Nakajima et al., 2020). Genomic DNA of a candidate strain, sensitive to G-418 and resistant to 5-FdU, was digested with *Sac*II, and transferred to a Nytran membrane (Cytiva, Tokyo, Japan). The blot was hybridized with a DIG-labeled *Tri6* probe, which was prepared using a PCR DIG Probe Synthesis Kit (Roche Diagnostics GmbH, Mannheim, Germany) and primers described in the figure (left panel). Predicted sizes of single bands were detected for the digested DNA (right panel).

1 TCGCGCGTTT CGGTGATGAC GGTGAAAACC TCTGACACAT GCAGCTCCCG GAGACGGTCA CAGCTTGTCT GTAAGCGGAT GCCGGGAGCA GACAAGCCCG

101 TCAGGGCGCG TCAGCGGGTG TTGGCGGGTG TCGGGGCTGG CTTAACTATG CGGCATCAGA GCAGATTGTA CTGAGAGTGC ACCATATGCG GTGTGAAATA

201 CCGCACAGAT GCGTAAGGAG AAAATACCGC ATCAGGCGCC ATTCGCCATT CAGGCTGCGC AACTGTTGGG AAGGGCGATC GGTGCGGGCC TCTTCGCTAT

CjTri6pro_S

301 TACGCCAGCT GGCGAAAGGG GGATGTGCTG CAAGGCGATT AAGTTGGGTA ACGCCAGGGT TTTCCCAGTC ACGACGTTGT AAAACGACGG CCAGTGAATT

Tri6 Upstream Region

CjTri6pro_S

401 CGAGCTCGGT ACCCAGGTAC CTTGTCTATC GCGTCTCTAG AGTGCTTTGC ATGCGTTGTG GCCGTAAACG CTCACAACTC TGAAGTTGTC CTCAGTATCG

Tri6 Upstream Region

501 CCGTGTCAGA TAAGCTTCGC AAGAGTGTCC GGTCGGAAAC TCACCAATCA ACTCAGCAGG ATGAACAAGG GGTCTGAAAG GCCTGGCAGG CCTGACAGGA

Tri6 Upstream Region

601 GTGATAAAAT GTGAGAAGAG ATATGCCGAT ACAACCGTGT AACTTGTGAA ACGGGGCATG GAATCCCATG GCAAGTTATG GGGTCAGCAG CAACTGAATT

Tri6 Upstream Region

701 GCCTACGAGT CAAGAAGTGC ATCCTTTCAC CGGCGGCTTA TCCGAAGTTG CTGCCGATCA GATACAGACA TGCATGCAGA GTGGTACGAC TGCGCGGAAG

Tri6 Upstream Region

801 AATAAGAATC ATCAGTGCGC CGCAATGTTA AAAACTGATG TGCGGAAGCA ACATTAAGCT TTGGAGGCAT GCCAGGGTCT TGTCTCGAAA TATCTTTGTC

Tri6 Upstream Region

901 TACCGAGACC CATGCATGGT CAAAAGTATG TACATGGATG GTCTTGCACA GAAGACAGCC TCGAGTGTTA TGCAGACTGT CACGGCTGCA GTAAGTTGCC

Tri6 Upstream Region

1001 ACAGACTCGA ATCGATTATC ATTGACCGTT CGGAAGCGCT CTGTTAGGAA TCTTTCTAGA CCACAACTAC CACTTTGGCA TCTGCATACT AACACTAGTA

Tri6 Upstream Region

1101 GCCACATAGT AAACCTTCAA CTGCCGCCGC ATCAAACTGT AAACAGGTAC CGGCCGACGC GTCTCGGATA AGAATACCTT TTAAACTGCC GTAGCAAACT

Tri6 Upstream Region

1201 GTAATTGTCG GTACTTCTCG GACAATATTT TCATGGCTTT CAGAAGCTTT CACTTTTAAT AAAACTTGAT CTGAATAAGA AACTTTATCA ATCGTATCCC

Tri6 Upstream Region

Tri6nsm

CjTri6_M4Um_S

M I Y *

1301 ATCCCATCAA GGCTCAAGCC ATCTTTTATT TTTTATTTTT TGCATCGCCA ACCAATATAT TGAACATCTA TTTTGACTAC CCTCGAAATG ATTTACTAGG

CjTri6pro_AS

nonsense mutation

Tri6nsm

CjTri6_M4Um_S

1401 AGGCCGAATC TCACTACGAA TCTTGGAGCG CCTTGCCCCT CTTTGATCGA GTTGCGTCTC CCGATCCTGC CAAGGACTTT GTCCCAGATC TAAACGACTA

Tri6nsm

1501 TGAATCACCA ACATTCGAAA TAGATCTTCT CTCAGAAACT TATGACTTTG ACAACTTCCC CACATACTCT CTACCAACGG TGGATTCAAC CAAGACTTTG

Tri6nsm

1601 TACTCCGAAG AACCACTTGT TTGCTTCGAC TTTGACTTCG CGAACCCGGC TATCGAAAAT TATATAACCA CATCGTCGGG ACTGTTGGAC GCAGTGCCAA

Tri6nsm

1701 GCCAGCTTAT CGCCCTTCCC ACCTTCACAC GGCCAAGCAA ATGCCCATTC CCTAGTTGCA AGTCGGCCAC AGTCTTTGAA AGCGGACGGG ACTTTAGGCG

Tri6nsm

1801 GCATTACCGG CAACACTTCA AGCGCTTTTT CTGTCGCTAC TCAGAATGCC CTCAGTCAGC TCAAGACCTG CAAGAAGTCG GCACCAAAGG CTTTGCGACT

Tri6nsm

1901 CGCAAGGACC GTGCTCGGCA TGAGTCTAAG CACAAACCAA CAGTGCGGTG CCCTTGGCAA GACAAGGAAG GACAACAATG TCTGAGGGTC TTTAGCAGGG

Tri6 downstream region

Tri6nsm

2001 TGGATAACAT GCGAGATCAC TATAGGCGGA TACATAAGTG TTGACGAGGG ATCGGTGTGC AAAACTGCAG ATAGTTACTC ATAAAAGGCA ACATTTGCGA

Tri6 downstream region

2101 AAATCAATAA CAATATCTCC ATAAATATCC CATTAGACTT TTTGAGTTTC ATAAATGATG ACGAGTTTGG CGCCGATCGA TGCTGTTTCC AGCACCAATT

Tri6 downstream region

2201 CATAATATAC TCCATAGTAG CCGAGACCCT GCAGCTCATT GGTACCAGCC CTGTTTTTAT TGCACAATTA ATGACACTCT ATTCTGCACG CGACTCAACG

Tri6 downstream region

2301 GCTTTCAACA CTTACATATT GCCTCTACGC GTGGCTGTGT CTAAGCGAGA TCAGCGCGGT CATTGTTATT GCTGCGTTGC TTTGGCCCCG TACGTTCCAC

Tri6 downstream region

2401 AACAATCTGG CCACTGCAAG GTATTTGCGG CCGCTGGTTA AATTCTCTTA GTAGTCTGCC TCATTTGGCA GGTGCCAATA ATATATTTTA CCCGCGTTTG

Tri6 downstream region

2501 GCTGGCCATG CTACCCAAAA GGCTTCATAC ATATCTGGCC CATATCGTTT ACTCGGTTCC ACGTTTACGA GCATACGCCT CTCGTATCAA TGTCGCAAAC

Tri6 downstream region

2601 AAACATACGT CATTGGTTAC TATGCCGGGT TTCCGCGAAC CATTGCTGTG CCGCAAAAGG ATGCATCGGC ACTCTAAAGA TTGCGATCCT GTGTTAGAGG

Tri6 downstream region

2701 GTGACTTGAG TCAAGAAGTA ACGTAACAAG TGAGATAATG AAATTAATTA CCTGAGGGCA ATTTAAGGTT TCAACCTCCG AGGAAAGCCA TTCGTCGTGG

Tri6 downstream region
2801 GATATAATCT GCAATAGGGT AGGCTTTGCT GTTTTTTTCG GAGCATTTGT GACTGTTTTG GATTGTGTCT TTGGGGTCCT TTTGCTTTCA AGGCTGGTGA

Tri6 downstream region
2901 GTTTGCAAGG AACGAGTCAG TCACGGCAGG CACTGAGTCA AACACTGTTT CGTAATAATC ATGATTTGTT GTTGCAAAAT TCCAACGAAG CTTTGCGCGG

Tri6 downstream region
3001 TCAGTCAAAT TCGCACACGT CGATACTTTT TGCTGGCATT GGCCCAGTAT TCATCCATGA GTGGCTGAAC CGTAGTTGAT TCTCAAGTCC AACCCTAATA

Tri6 downstream region
3101 GTGCCCGGCG GAATGAGACG TTTTCGGGTG TCTGTAGCCG AGATGTGGAT AGTAACGGTA ACCCTAGTCA AATGAGACGT GGGCAGGGTT CATGGTTGTT

Tri6d ownstream region
3201 GAACCTTGTT CATCAGAATG TTGATGCCGT TGACCTACGG AATACCATCT TTCACATGTA TTTGTTCCCA ACCCACGTGG CTATACCAAC ATCCGCTGTA

Tri6 downstream region BamHI
3301 TATTCATTGG TTGGCTTTAT ATATTGATAC AGGTAATTTC AAGATCGCGA GCGGGAATTT CCTTGTCGGA TCAGAGAGAT GCTCTGGGGA TCCTCTAGAG
CompJTri6ter_AS

3401 TCGACCTGCA GGCATGCAAG CTTGGCGTAA TCATGGTCAT AGCTGTTTCC TGTGTGAAAT TGTTATCCGC TCACAATTCC ACACAACATA CGAGCCGGAA
3501 GCATAAAGTG TAAAGCCTGG GGTGCCTAAT GAGTGAGCTA ACTCACATTA ATTGCGTTGC GCTCACTGCC CGCTTTCCAG TCGGGAAACC TGTCGTGCCA
3601 GCTGCATTAA TGAATCGGCC AACGCGCGGG GAGAGGCGGT TTGCGTATTG GGCGCTCTTC CGCTTCCTCG CTCACTGACT CGCTGCGCTC GGTCGTTCGG
3701 CTGCGGCGAG CGGTATCAGC TCACTCAAAG GCGGTAATAC GGTTATCCAC AGAATCAGGG GATAACGCAG GAAAGAACAT GTGAGCAAAA GGCCAGCAAA
3801 AGGCCAGGAA CCGTAAAAAG GCCGCGTTGC TGGCGTTTTT CCATAGGCTC CGCCCCCCTG ACGAGCATCA CAAAAATCGA CGCTCAAGTC AGAGGTGGCG
3901 AAACCCGACA GGACTATAAA GATACCAGGC GTTTCCCCCT GGAAGCTCCC TCGTGCGCTC TCCTGTTCCG ACCCTGCCGC TTACCGGATA CCTGTCCGCC
4001 TTTCTCCCTT CGGGAAGCGT GGCGCTTTCT CAATGCTCAC GCTGTAGGTA TCTCAGTTCG GTGTAGGTCG TTCGCTCCAA GCTGGGCTGT GTGCACGAAC
4101 CCCCCGTTCA GCCCGACCGC TGCGCCTTAT CCGGTAACTA TCGTCTTGAG TCCAACCCGG TAAGACACGA CTTATCGCCA CTGGCAGCAG CCACTGGTAA
4201 CAGGATTAGC AGAGCGAGGT ATGTAGGCGG TGCTACAGAG TTCTTGAAGT GGTGGCCTAA CTACGGCTAC ACTAGAAGGA CAGTATTTGG TATCTGCGCT
4301 CTGCTGAAGC CAGTTACCTT CGGAAAAAGA GTTGGTAGCT CTTGATCCGG CAAACAAACC ACCGCTGGTA GCGGTGGTTT TTTTGTTTGC AAGCAGCAGA
4401 TTACGCGCAG AAAAAAAGGA TCTCAAGAAG ATCCTTTGAT CTTTTCTACG GGGTCTGACG CTCAGTGGAA CGAAAACTCA CGTTAAGGGA TTTTGGTCAT
4501 GAGATTATCA AAAAGGATCT TCACCTAGAT CCTTTTAAAT TAAAAATGAA GTTTTAAATC AATCTAAAGT ATATATGAGT AAACTTGGTC TGACAGTTAC
bl

4601 CAATGCTTAA TCAGTGAGGC ACCTATCTCA GCGATCTGTC TATTTCGTTC ATCCATAGTT GCCTGACTCC CCGTCGTGTA GATAACTACG ATACGGGAGG
bla

4701 GCTTACCATC TGGCCCCAGT GCTGCAATGA TACCGCGAGA CCCACGCTCA CCGGCTCCAG ATTTATCAGC AATAAACCAG CCAGCCGGAA GGGCCGAGCG
bla

4801 CAGAAGTGGT CCTGCAACTT TATCCGCCTC CATCCAGTCT ATTAATTGTT GCCGGGAAGC TAGAGTAAGT AGTTCGCCAG TTAATAGTTT GCGCAACGTT
bla

4901 GTTGCCATTG CTACAGGCAT CGTGGTGTCA CGCTCGTCGT TTGGTATGGC TTCATTCAGC TCCGGTTCCC AACGATCAAG GCGAGTTACA TGATCCCCCA
bl

5001 TGTTGTGCAA AAAAGCGGTT AGCTCCTTCG GTCCTCCGAT CGTTGTCAGA AGTAAGTTGG CCGCAGTGTT ATCACTCATG GTTATGGCAG CACTGCATAA
bla

5101 TTCTCTTACT GTCATGCCAT CCGTAAGATG CTTTTCTGTG ACTGGTGAGT ACTCAACCAA GTCATTCTGA GAATAGTGTA TGCGGCGACC GAGTTGCTCT
bla

5201 TGCCCGGCGT CAATACGGGA TAATACCGCG CCACATAGCA GAACTTTAAA AGTGCTCATC ATTGGAAAAC GTTCTTCGGG GCGAAAACTC TCAAGGATCT
bla

5301 TACCGCTGTT GAGATCCAGT TCGATGTAAC CCACTCGTGC ACCCAACTGA TCTTCAGCAT CTTTTACTTT CACCAGCGTT TCTGGGTGAG CAAAAACAGG
bl

5401 AAGGCAAAAT GCCGCAAAAA AGGGAATAAG GGCGACACGG AAATGTTGAA TACTCATACT CTTCCTTTTT CAATATTATT GAAGCATTTA TCAGGGTTAT
bla

5501 TGTCTCATGA GCGGATACAT ATTTGAATGT ATTTAGAAAA ATAAACAAAT AGGGGTTCCG CGCACATTTC CCCGAAAAGT GCCACCTGAC GTCTAAGAAA
5601 CCATTATTAT CATGACATTA ACCTATAAAA ATAGGCGTAT CACGAGGCCC TTTCGTC

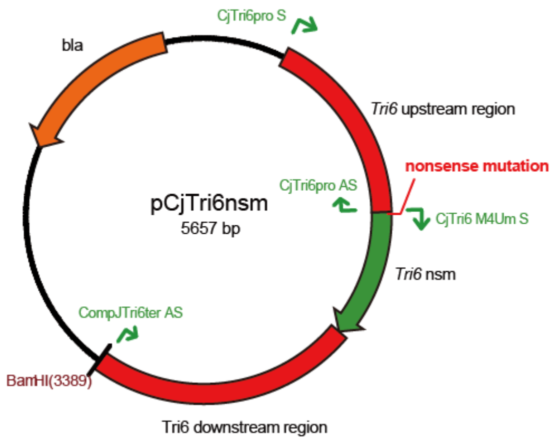

Supplementary Figure 5 | Sequence and structure of pCjTri6nsm. Two pairs of PCR primers (CjTri6pro_S: 5′-TCGAGCTCGGTACCCAGGTACCTTGTCTATCGCGT-3′ and CjTri6pro_AS: 5′-GTAAATCATTTCGAGGGTAGTCA-3′; CjTri6_M4Um_S: 5′-CTCGAAATGATTTACTAGGAGGCCGAATCTCACTACGA-3′ and CompJTri6ter_AS: 5′-CTCTAGAGGATCCCCAGAGCATCTCTCTGATCCGA-3′; sequences overlapping in pUC19 underlined), with 15 bp overhangs necessary for Gibson Assembly and a nonsense mutation for the gene inactivation (doubly underlined), were designed as shown in the figure. The two PCR fragments and *Sma*I-linearized pUC19 were assembled by Gibson Assembly.

Tri10 start codon

```
F. graminearum       ATGGATTTCCCAAAGCCTAGACAAGTCCGAGAGACGAGCCTGTTGATGTACTACCTAGACGTCGTGTTTCCTCTGCAATG  80
F. sporotrichioides  ATGGAATTTCCGAAGCCGAGACAGTTCAGAGAGACGAGTCTGTTGATGTACTACCTGGACGTCGTGTTTCCTCTGCAATA  80

F. graminearum       CATCAACCCAAACAACAATTGTCTGGGAAAGAGAGAGTGGCTGTTGACTATACTGACCTCTGCGCGGCCTACGTACTATG  160
F. sporotrichioides  CATTTCACCAAACAACAATTGTCTGGGGAAAAGAGAGTGGTTGTTGACTATACTAACTTCTGCTCGCCCTACATACTATG  160

F. graminearum       CCACATTGTGCATGTCGCTCCTCTATAAAGAATCGCTTTCAAGCCCTTGCAGATCTGAACAGGCGATGGTATGGAAGAGA  240
F. sporotrichioides  CAACATTGTGCCTGGCCCTCCTTTATAAAGAGTCTCTTTCAACCTCGTGCAGAAGTGAACAAACATTAGTATGGAAGAGG  240

F. graminearum       GAGAAGACATACTACTACATTCTTGCACTCCAGGAGTCTCAGAAGCTGCTGGGTGGGCTCGACAAGACATTTGGCATCAC  320
F. sporotrichioides  GAAAAGACCTACTACTACATTCTTGCGCTCCAGGAGTCTCAAAAGCTCTTGGGTGGGCTTAACAAGACCTTTGGTATCAC  320

F. graminearum       AAGGCTGAAAGGTACCGTCGTTGCCCTTGCTTGCATGCTACAGCTTATCAGTTTTGAGGTAAGACGAATCCACCATTGTT  400
F. sporotrichioides  AAGGCTGAAAGGGACGGTCGTTGCCCTCGCTTGCATGCTTCAGCTTATCGGGTTTGAGGTAAGACGAATCCACCATGACT  400

F. graminearum       TCGATGCTCGATGTCGATGCTCGATATCCGATCTACGATTATCGTTGGTCACTAACAAATTAAAATAGTCTTCGCACCTA  480
F. sporotrichioides  A-------------CGATGTTCAATACCAGATGTATAATTATTGTTGGCGACTAACGCATTGCGACAGTCTTCGCACCTG  467

F. graminearum       AGCAGGGGAGATTGGCGCGTTCACCTCCATGCGGCCAACATACTCATTCCTGTCTTGGTTGAGGGATGGTCCACAGCTTT  560
F. sporotrichioides  AGTAGGGGAGATTGGCGTGTTCACCTCCTTGCTGCCAACACACTCATTCCTGTGTTGGCCGAGGGTTGGTCCACAGCTTT  547

F. graminearum       GCAATCAGGCCCCCCAGCCACCTCCATATGGTGCGAGCTGGATGAATCACACTTCGGCTCGACTGAAGATCAAACCTCTT  640
F. sporotrichioides  GCAGTCAGGCCCCCCAGCCACTTCCATATGGTGTGAGTTAGACGAATCGGACTTTGATTCAATCGACGATCAGACCTCTT  627

F. graminearum       TGAGCTTCGAATACGTCGGAGCTTTGAGATTCCTGTCAAACTCACTCGCCGCAGTCGGCATCCTGTCTTGCATATCTATT  720
F. sporotrichioides  TGAGCTTCGAATATCTCGGAGCTTTGAGATTCCTGTCGAACTCCTTGGCGAAAATCGGCATCTTATCTTGCATATCTGTT  707

F. graminearum       GGCCCATCAGCACCATTTGAAGATTACGGCCATCTCCTGGACCAGCCAGGCCTTATACAGCTGGACGAGGTGCTGGGGTG  800
F. sporotrichioides  GGTCCAGCAGCGCCATTTGAAGACTATGGTCACCTCCTGGACCAGCCAGGCCTGATACAGCTGGAAGAGGTACTGGGGTG  787

F. graminearum       CAGGAATTGGACCATGTTGACTATTCTCGAAGTGGGTAAGCTGGATCGTTGGAAGCGACAGGAGCAAGAACATAATCGCT  880
F. sporotrichioides  CAAGAACTGGGCCATGCTGACTATTCTTGAAGTGGGTAAGCTGGACAGGTGGAAGCGCCAGGAGCAGGAACATAACCGTT  867

F. graminearum       TGAGCCTAAAGACGCTCGCTAGGCGCGCCATGATGATTGAGGATATGTTGTCAGACGAGCTACAAAGGCTACCGACAGAC  960
F. sporotrichioides  TGAGCCTGAAGACACTTGCTATGCGCGCAATGATTATAGAGGATATGTTGACAGACGAACTACAAAAACTTCCGACAAGC  947

F. graminearum       GAGACGCTTCCAGACCTCATCACTCAGATTTACGCCGCCTCTATCATGACGTATCTGCATACAGTAGTTTCCGGACTCAA  1040
F. sporotrichioides  GAGACGCTACCGGATCTGATCACCCACATTTACGCCGCCTCTATCGCGACATACCTGCATACAGTAGTTTCAGGACTGAA  1027

F. graminearum       TCCCAACCTTTCAGAGGTTCAGGATAGTGTGGCCGGGACGCTTCAATTGTTGGAGAGGCTCCCAAATCTTGAAGCTGTCA  1120
F. sporotrichioides  TCCCAACCTTTCAGAGGTCCAGGATAGCGTGTGCGCAACAATATTATTGTTGGAGAGGCTCCCAGACTTGCAAGCTGTCG  1107

F. graminearum       CGAGCGTTACTTGGCCTCTAGCTGTCACAGGATGCATGGCCTCAGAAAGTCATAAGGACTTTTTCAGAAATACTCTGAGG  1200
F. sporotrichioides  CGAGCGTTACTTGGCCTTTGGCTGTCACGGGGTGTATGGCTTCAGAAAGTCATAAGGACTTTTTCAGAAGTACTCTGAGG  1187

F. graminearum       TCGTATGAGGCGACATTCAGCTCCTTAAAAAAGTATGACGGAACTCTTCAGGTCTTGGAAGACGCTTGGAAGAGAAGAGA  1280
F. sporotrichioides  TCTTATGAAGCGACATTCAGCTCGTTAAAGAAGTACGACGGAGTTCTTGAGGTGTTGGAAGATGCTTGGAAGAAAAGAGA  1267

F. graminearum       GATAGATACAGAGTCTCCAATGAGATGGGAAGACTTGACGGATCACCATGGGCTTCCAGTGCTACTTTGGTAG  1353
F. sporotrichioides  GGTAGATACAGAGTCTCCAATGAGGTGGGAGGATTTGATGGATCACCATGGGCTTCCAGTGCTCCTTTTCTAA  1340
```

Supplementary Figure 6 | Sequence alignment of *Tri10* gene from *F. graminearum* PH-1 (NC_026475 REGION: complement [6646050..6647402]) and *F. sporotrichioides* NRRL 3299 (AF364179 REGION: 2170..3509). Tri6p-binding consensus sequences YNAGGCC on the coding strand (shaded in red) and non-coding strand (boxed in blue) are shown. *F. graminearum* contains four Tri6p-binding consensus sequences while *F. sporotrichioides* contains three.

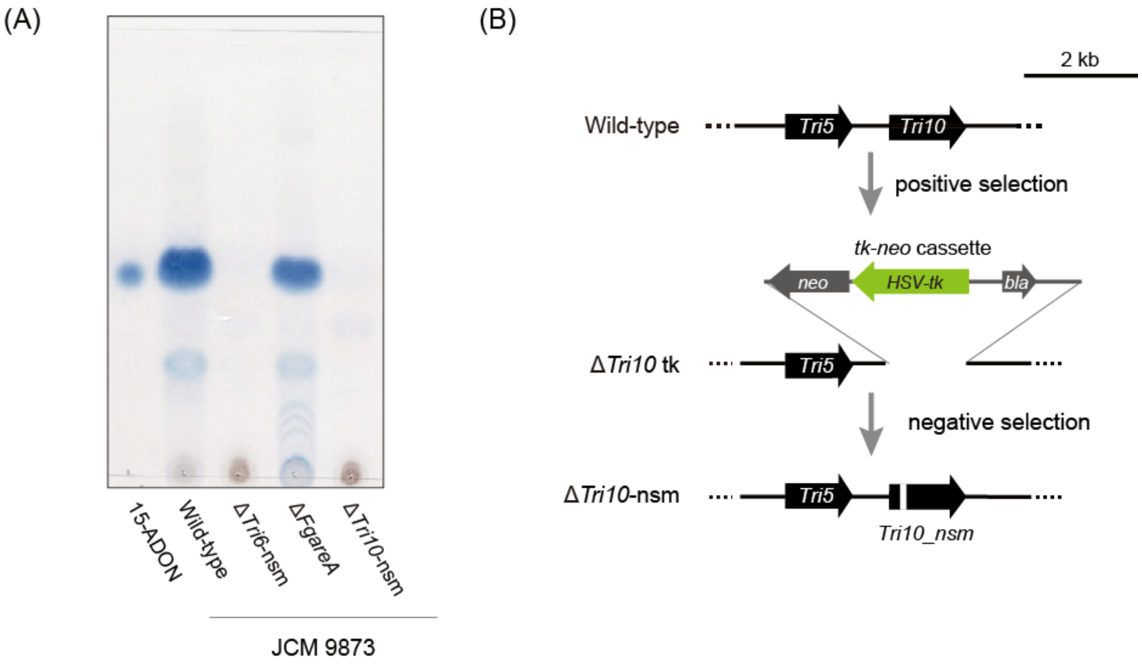

Supplementary Figure 7 | Toxin production assays of the various mutant strains of *F. graminearum* JCM 9873. (A) Each of the four strains analyzed was cultured on the defined media, pH 2.5, with L-glutamine as the nitrogen source and sucrose as the carbon source (**Supplementary Table 1**) for 8 days. The TLC panel shows the spots of 15-ADON extracted from 500 μL of the medium of each culture with ethyl acetate. (B) Schematic representation of the genomic structure Δ*Tri10*-nsm mutant strain. See **Supplementary Figure 8** for experimental details of the mutant strain construction and confirmation. The genomic structure of the Δ*FgareA* strain was described in a previous report (Nakajima et al., 2020). The genomic structure of Δ*Tri6*-nsm is shown in **Supplementary Figure 3E**.

(A)

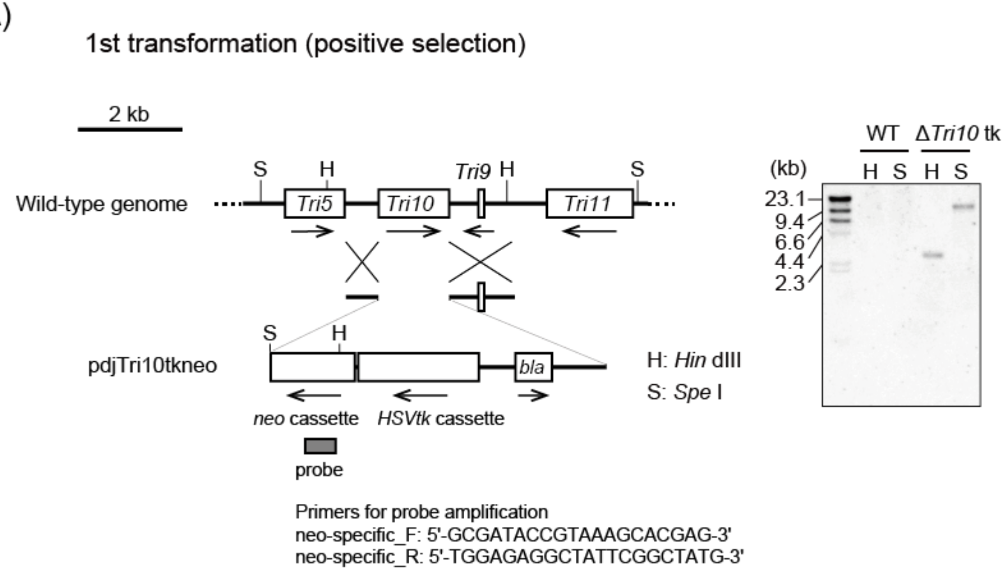

(B)

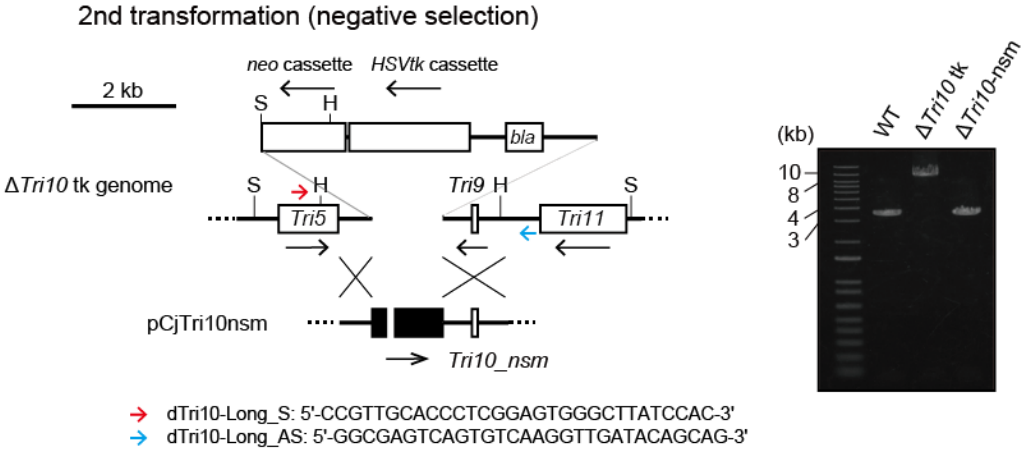

Supplementary Figure 8 | Generation of the Δ*Tri10* tk and Δ*Tri10*-nsm mutant strains. (A) Generation and Southern blot verification of the Δ*Tri10* tk strain. Strain JCM 9873 was transformed with pdjTri10tkneo (**Supplementary Figure 9**) and selected with hygromycin B (left panel). Genomic DNAs of the wild-type (WT) and candidate *Tri10* disruptant were digested with *Hind*III and *Spe*I, and then hybridized with a DIG-labeled probe (*neo*), which was prepared using a PCR DIG Probe Synthesis Kit (Roche Diagnostics GmbH, Mannheim, Germany) and primers described in the figure. Predicted sizes of single bands were detected for the *Hind*III and *Spe*I digested DNA

(right panel). (B) Generation and PCR verification of the Δ*Tri10*-nsm strain. Strain Δ*Tri10* tk was transformed with pCjTri10nsm (**Supplementary Figure 9**) and a candidate strain was obtained by conditional negative selection with 5-FdU (left panel). PCR was performed on the genomic DNAs of the WT, Δ*Tri10* tk, and Δ*Tri10*-nsm strains using primers (red and blue) located outside of the homologous region. Expected sizes of amplicons were obtained (right panel).

neo

1 AATTCAGAGC CCATGGGATC GGCCATTGAA CAAGATGGAT TGCACGCAGG TTCTCCGGCC GCTTGGGTGG AGAGGCTATT CGGCTATGAC TGGGCACAAC
TTAAGTCTCG GGTACCCTAG CCGGTAACTT GTTCTACCTA ACGTGCGTCC AAGAGGCCGG CGAACCCACC TCTCCGATAA GCCGATACTG ACCCGTGTTG

neo

101 AGACAATCGG CTGCTCTGAT GCCGCCGTGT TCCGGCTGTC AGCGCAGGGG CGCCCGGTTC TTTTTGTCAA GACCGACCTG TCCGGTGCCC TGAATGAACT
TCTGTTAGCC GACGAGACTA CGGCGGCACA AGGCCGACAG TCGCGTCCCC GCGGGCCAAG AAAAACAGTT CTGGCTGGAC AGGCCACGGG ACTTACTTGA

neo

201 GCAGGACGAG GCAGCGCGGC TATCGTGGCT GGCCACGACG GGCGTTCCTT GCGCAGCTGT GCTCGACGTT GTCACTGAAG CGGGAAGGGA CTGGCTGCTA
CGTCCTGCTC CGTCGCGCCG ATAGCACCGA CCGGTGCTGC CCGCAAGGAA CGCGTCGACA CGAGCTGCAA CAGTGACTTC GCCCTTCCCT GACCGACGAT

neo

301 TTGGGCGAAG TGCCGGGGCA GGATCTCCTG TCATCTCACC TTGCTCCTGC CGAGAAAGTA TCCATCATGG CTGATGCAAT GCGGCGGCTG CATACGCTTG
AACCCGCTTC ACGGCCCCGT CCTAGAGGAC AGTAGAGTGG AACGAGGACG GCTCTTTCAT AGGTAGTACC GACTACGTTA CGCCGCCGAC GTATGCGAAC

neo

401 ATCCGGCTAC CTGCCCATTC GACCACCAAG CGAAACATCG CATCGAGCGA GCACGTACTC GGATGGAAGC CGGTCTTGTC GATCAGGATG ATCTGGACGA
TAGGCCGATG GACGGGTAAG CTGGTGGTTC GCTTTGTAGC GTAGCTCGCT CGTGCATGAG CCTACCTTCG GCCAGAACAG CTAGTCCTAC TAGACCTGCT

neo

501 AGAGCATCAG GGGCTCGCGC CAGCCGAACT GTTCGCCAGG CTCAAGGCGC GCATGCCCGA CGGCGAGGAT CTCGTCGTGA CCCATGGCGA TGCCTGCTTG
TCTCGTAGTC CCCGAGCGCG GTCGGCTTGA CAAGCGGTCC GAGTTCCGCG CGTACGGGCT GCCGCTCCTA GAGCAGCACT GGGTACCGCT ACGGACGAAC

neo

601 CCGAATATCA TGGTGGAAAA TGGCCGCTTT TCTGGATTCA TCGACTGTGG CCGGCTGGGT GTGGCGGACC GCTATCAGGA CATAGCGTTG GCTACCCGTG
GGCTTATAGT ACCACCTTTT ACCGGCGAAA AGACCTAAGT AGCTGACACC GGCCGACCCA CACCGCCTGG CGATAGTCCT GTATCGCAAC CGATGGGCAC

neo

701 ATATTGCTGA AGAGCTTGGC GGCGAATGGG CTGACCGCTT CCTCGTGCTT TACGGTATCG CCGCTCCCGA TTCGCAGCGC ATCGCCTTCT ATCGCCTTCT
TATAACGACT TCTCGAACCG CCGCTTACCC GACTGGCGAA GGAGCACGAA ATGCCATAGC GGCGAGGGCT AAGCGTCGCG TAGCGGAAGA TAGCGGAAGA

neo TrpC terminator

801 TGACGAGTTC TTCTGACGGG CTGGCTGCAG TGGTGGGGGA TCCACTTAAC GTTACTGAAA TCATCAAACA GCTTGACGAA TCTGGATATA AGATCGTTGG
ACTGCTCAAG AAGACTGCCC GACCGACGTC ACCACCCCCT AGGTGAATTG CAATGACTTT AGTAGTTTGT CGAACTGCTT AGACCTATAT TCTAGCAACC

TrpC terminator

901 TGTCGATGTC AGCTCCGGAG TTGAGACAAA TGGTGTTCAG GATCTCGATA AGATACGTTC ATTTGTCCAA GCAGCAAAGA GTGCCTTCTA GTGATTTAAT
ACAGCTACAG TCGAGGCCTC AACTCTGTTT ACCACAAGTC CTAGAGCTAT TCTATGCAAG TAAACAGGTT CGTCGTTTCT CACGGAAGAT CACTAAATTA

TrpC terminator

1001 AGCTCCATGT CAACAAGAAT AAAACGCGTT TCGGGTTTAC CTCTTCCAGA TACAGCTCAT CTGCAATGCA TTAATGCATT GGACCTCGCA ACCCTAGTAC
TCGAGGTACA GTTGTTCTTA TTTTGCGCAA AGCCCAAATG GAGAAGGTCT ATGTCGAGTA GACGTTACGT AATTACGTAA CCTGGAGCGT TGGGATCATG

TrpC terminator

1101 GCCCTTCAGG CTCCGGCGAA GCAGAAGAAT AGCTTAGCAG AGTCTATTTT CATTTTCGGG AGACGAGATC AAGCAGATCA ACGGTCGTCA AGAGACCTAC
CGGGAAGTCC GAGGCCGCTT CGTCTTCTTA TCGAATCGTC TCAGATAAAA GTAAAAGCCC TCTGCTCTAG TTCGTCTAGT TGCCAGCAGT TCTCTGGATG

TrpC terminator

1201 GAGACTGAGG AATCCGCTCT TGGCTCCACG CGACTATATA TTTGTCTCTA ATTGTACTTT GACATGCTCC TCTTCTTTAC TCTGATAGCT TGACTATGAA
CTCTGACTCC TTAGGCGAGA ACCGAGGTGC GCTGATATAT AAACAGAGAT TAACATGAAA CTGTACGAGG AGAAGAAATG AGACTATCGA ACTGATACTT

TrpC terminator

1301 AATTCCGTCA CCAGCCCCTG GGTTCGCAAA GATAATTGCA CTGTTTCTTC CTTGAACTCT CAAGCCTACA GGACACACAT TCATCGTAGG TATAAACCTC
TTAAGGCAGT GGTCGGGGAC CCAAGCGTTT CTATTAACGT GACAAAGAAG GAACTTGAGA GTTCGGATGT CCTGTGTGTA AGTAGCATCC ATATTTGGAG

TrpC terminator

1401 GAAAATCATT CCTACTAAGA TGGGTATACA ATAGTAACCA TGGTTGCCTA GTGAATGCTC CGTAACACCC AATACGCCGG CCGAAACTTT TTTACAACTC
CTTTTAGTAA GGATGATTCT ACCCATATGT TATCATTGGT ACCAACGGAT CACTTACGAG GCATTGTGGG TTATGCGGCC GGCTTTGAAA AAATGTTGAG

TrpC terminator SpeI

1501 TCCTATGAGT CGTTTACCCA GAATGCACAG GTACACTTGT TTAGAGGTAA TCCTTCTTTC TAGAGGATCC ACTAGTGCGT TAACATCATA TGCCAGATCT
AGGATACTCA GCAAATGGGT CTTACGTGTC CATGTGAACA AATCTCCATT AGGAAGAAAG ATCTCCTAGG TGATCACGCA ATTGTAGTAT ACGGTCTAGA

SacII

1601 AAGCGGCCGC CACCGCGGTG GAGCTCCAGC TTTTGTTCCC TTTAGTGAGG GTTAATTTCG AGCTTGGCGT AATCATGGTC ATAGCTGTTT CCTGTGTGAA
TTCGCCGGCG GTGGCGCCAC CTCGAGGTCG AAAACAAGGG AAATCACTCC CAATTAAAGC TCGAACCGCA TTAGTACCAG TATCGACAAA GGACACACTT

1701 ATTGTTATCC GCTCACAATT CCACACAACA TACGAGCCGG AAGCATAAAG TGTAAAGCCT GGGGTGCCTA ATGAGTGAGC TAACTCACAT TAATTGCGTT
TAACAATAGG CGAGTGTTAA GGTGTGTTGT ATGCTCGGCC TTCGTATTTC ACATTTCGGA CCCCACGGAT TACTCACTCG ATTGAGTGTA ATTAACGCAA

1801 GCGCTCACTG CCCGCTTTCC AGTCGGGAAA CCTGTCGTGC CAGCTGCATT AATGAATCGG CCAACGCGCG GGGAGAGGCG GTTTGCGTAT TGGGCGCTCT
CGCGAGTGAC GGGCGAAAGG TCAGCCCTTT GGACAGCACG GTCGACGTAA TTACTTAGCC GGTTGCGCGC CCCTCTCCGC CAAACGCATA ACCCGCGAGA

1901 TCCGCTTCCT CGCTCACTGA CTCGCTGCGC TCGGTCGTTC GGCTGCGGCG AGCGGTATCA GCTCACTCAA AGGCGGTAAT ACGGTTATCC ACAGAATCAG
AGGCGAAGGA GCGAGTGACT GAGCGACGCG AGCCAGCAAG CCGACGCCGC TCGCCATAGT CGAGTGAGTT TCCGCCATTA TGCCAATAGG TGTCTTAGTC

2001 GGGATAACGC AGGAAAGAAC ATGTGAGCAA AAGGCCAGCA AAAGGCCAGG AACCGTAAAA AGGCCGCGTT GCTGGCGTTT TTCCATAGGC TCCGCCCCCC
CCCTATTGCG TCCTTTCTTG TACACTCGTT TTCCGGTCGT TTTCCGGTCC TTGGCATTTT TCCGGCGCAA CGACCGCAAA AAGGTATCCG AGGCGGGGGG

2101 TGACGAGCAT CACAAAAATC GACGCTCAAG TCAGAGGTGG CGAAACCCGA CAGGACTATA AAGATACCAG GCGTTTCCCC CTGGAAGCTC CCTCGTGCGC
ACTGCTCGTA GTGTTTTTAG CTGCGAGTTC AGTCTCCACC GCTTTGGGCT GTCCTGATAT TTCTATGGTC CGCAAAGGGG GACCTTCGAG GGAGCACGCG

2201 TCTCCTGTTC CGACCCTGCC GCTTACCGGA TACCTGTCCG CCTTTCTCCC TTCGGGAAGC GTGGCGCTTT CTCATAGCTC ACGCTGTAGG TATCTCAGTT
AGAGGACAAG GCTGGGACGG CGAATGGCCT ATGGACAGGC GGAAAGAGGG AAGCCCTTCG CACCGCGAAA GAGTATCGAG TGCGACATCC ATAGAGTCAA

2301 CGGTGTAGGT CGTTCGCTCC AAGCTGGGCT GTGTGCACGA ACCCCCCGTT CAGCCCGACC GCTGCGCCTT ATCCGGTAAC TATCGTCTTG AGTCCAACCC
GCCACATCCA GCAAGCGAGG TTCGACCCGA CACACGTGCT TGGGGGGCAA GTCGGGCTGG CGACGCGGAA TAGGCCATTG ATAGCAGAAC TCAGGTTGGG

2401 GGTAAGACAC GACTTATCGC CACTGGCAGC AGCCACTGGT AACAGGATTA GCAGAGCGAG GTATGTAGGC GGTGCTACAG AGTTCTTGAA GTGGTGGCCT
CCATTCTGTG CTGAATAGCG GTGACCGTCG TCGGTGACCA TTGTCCTAAT CGTCTCGCTC CATACATCCG CCACGATGTC TCAAGAACTT CACCACCGGA

2501 AACTACGGCT ACACTAGAAG GACAGTATTT GGTATCTGCG CTCTGCTGAA GCCAGTTACC TTCGGAAAAA GAGTTGGTAG CTCTTGATCC GGCAAACAAA
TTGATGCCGA TGTGATCTTC CTGTCATAAA CCATAGACGC GAGACGACTT CGGTCAATGG AAGCCTTTTT CTCAACCATC GAGAACTAGG CCGTTTGTTT

2601 CCACCGCTGG TAGCGGTGGT TTTTTTGTTT GCAAGCAGCA GATTACGCGC AGAAAAAAAG GATCTCAAGA AGATCCTTTG ATCTTTTCTA CGGGGTCTGA
GGTGGCGACC ATCGCCACCA AAAAAACAAA CGTTCGTCGT CTAATGCGCG TCTTTTTTTC CTAGAGTTCT TCTAGGAAAC TAGAAAAGAT GCCCCAGACT

2701 CGCTCAGTGG AACGAAAACT CACGTTAAGG GATTTTGGTC ATGAGATTAT CAAAAAGGAT CTTCACCTAG ATCCTTTTAA ATTAAAAATG AAGTTTTAAA
GCGAGTCACC TTGCTTTTGA GTGCAATTCC CTAAAACCAG TACTCTAATA GTTTTTCCTA GAAGTGGATC TAGGAAAATT TAATTTTTAC TTCAAAATTT

```
2801  TCAATCTAAA GTATATATGA GTAAACTTGG TCTGACAGTT ACCAATGCTT AATCAGTGAG GCACCTATCT CAGCGATCTG TCTATTTCGT TCATCCATAG
      AGTTAGATTT CATATATACT CATTTGAACC AGACTGTCAA TGGTTACGAA TTAGTCACTC CGTGGATAGA GTCGCTAGAC AGATAAAGCA AGTAGGTATC
                                                                     bla
2901  TTGCCTGACT CCCCGTCGTG TAGATAACTA CGATACGGGA GGGCTTACCA TCTGGCCCCA GTGCTGCAAT GATACCGCGA GACCCACGCT CACCGGCTCC
      AACGGACTGA GGGGCAGCAC ATCTATTGAT GCTATGCCCT CCCGAATGGT AGACCGGGGT CACGACGTTA CTATGGCGCT CTGGGTGCGA GTGGCCGAGG
                                                    bla
3001  AGATTTATCA GCAATAAACC AGCCAGCCGG AAGGGCCGAG CGCAGAAGTG GTCCTGCAAC TTTATCCGCC TCCATCCAGT CTATTAATTG TTGCCGGGAA
      TCTAAATAGT CGTTATTTGG TCGGTCGGCC TTCCCGGCTC GCGTCTTCAC CAGGACGTTG AAATAGGCGG AGGTAGGTCA GATAATTAAC AACGGCCCTT
                                                    bla
3101  GCTAGAGTAA GTAGTTCGCC AGTTAATAGT TTGCGCAACG TTGTTGCCAT TGCTACAGGC ATCGTGGTGT CACGCTCGTC GTTTGGTATG GCTTCATTCA
      CGATCTCATT CATCAAGCGG TCAATTATCA AACGCGTTGC AACAACGGTA ACGATGTCCG TAGCACCACA GTGCGAGCAG CAAACCATAC CGAAGTAAGT
                                                    bla
3201  GCTCCGGTTC CCAACGATCA AGGCGAGTTA CATGATCCCC CATGTTGTGC AAAAAAGCGG TTAGCTCCTT CGGTCCTCCG ATCGTTGTCA GAAGTAAGTT
      CGAGGCCAAG GGTTGCTAGT TCCGCTCAAT GTACTAGGGG GTACAACACG TTTTTTCGCC AATCGAGGAA GCCAGGAGGC TAGCAACAGT CTTCATTCAA
                                                    bla
3301  GGCCGCAGTG TTATCACTCA TGGTTATGGC AGCACTGCAT AATTCTCTTA CTGTCATGCC ATCCGTAAGA TGCTTTTCTG TGACTGGTGA GTACTCAACC
      CCGGCGTCAC AATAGTGAGT ACCAATACCG TCGTGACGTA TTAAGAGAAT GACAGTACGG TAGGCATTCT ACGAAAAGAC ACTGACCACT CATGAGTTGG
                                                    bla
3401  AAGTCATTCT GAGAATAGTG TATGCGGCGA CCGAGTTGCT CTTGCCCGGC GTCAATACGG GATAATACCG CGCCACATAG CAGAACTTTA AAAGTGCTCA
      TTCAGTAAGA CTCTTATCAC ATACGCCGCT GGCTCAACGA GAACGGGCCG CAGTTATGCC CTATTATGGC GCGGTGTATC GTCTTGAAAT TTTCACGAGT
                                                    bla
3501  TCATTGGAAA ACGTTCTTCG GGGCGAAAAC TCTCAAGGAT CTTACCGCTG TTGAGATCCA GTTCGATGTA ACCCACTCGT GCACCCAACT GATCTTCAGC
      AGTAACCTTT TGCAAGAAGC CCCGCTTTTG AGAGTTCCTA GAATGGCGAC AACTCTAGGT CAAGCTACAT TGGGTGAGCA CGTGGGTTGA CTAGAAGTCG
                                                    bla
3601  ATCTTTTACT TTCACCAGCG TTTCTGGGTG AGCAAAAACA GGAAGGCAAA ATGCCGCAAA AAAGGGAATA AGGGCGACAC GGAAATGTTG AATACTCATA
      TAGAAAATGA AAGTGGTCGC AAAGACCCAC TCGTTTTTGT CCTTCCGTTT TACGGCGTTT TTTCCCTTAT TCCCGCTGTG CCTTTACAAC TTATGAGTAT
                                                    bla
3701  CTCTTCCTTT TTCAATATTA TTGAAGCATT TATCAGGGTT ATTGTCTCAT GAGCGGATAC ATATTTGAAT GTATTTAGAA AAATAAACAA ATAGGGGTTC
      GAGAAGGAAA AAGTTATAAT AACTTCGTAA ATAGTCCCAA TAACAGAGTA CTCGCCTATG TATAAACTTA CATAAATCTT TTTATTTGTT TATCCCCAAG
3801  CGCGCACATT TCCCCGAAAA GTGCCACCTA AATTGTAAGC GTTAATATTT TGTTAAAATT CGCGTTAAAT TTTTGTTAAA TCAGCTCATT TTTTAACCAA
      GCGCGTGTAA AGGGGCTTTT CACGGTGGAT TTAACATTCG CAATTATAAA ACAATTTTAA GCGCAATTTA AAAACAATTT AGTCGAGTAA AAAATTGGTT
3901  TAGGCCGAAA TCGGCAAAAT CCCTTATAAA TCAAAAGAAT AGACCGAGAT AGGGTTGAGT GTTGTTCCAG TTTGGAACAA GAGTCCACTA TTAAAGAACG
      ATCCGGCTTT AGCCGTTTTA GGGAATATTT AGTTTTCTTA TCTGGCTCTA TCCCAACTCA CAACAAGGTC AAACCTTGTT CTCAGGTGAT AATTTCTTGC
4001  TGGACTCCAA CGTCAAAGGG CGAAAAACCG TCTATCAGGG CGATGGCCCA CTACGTGAAC CATCACCCTA ATCAAGTTTT TTGGGGTCGA GGTGCCGTAA
      ACCTGAGGTT GCAGTTTCCC GCTTTTTGGC AGATAGTCCC GCTACCGGGT GATGCACTTG GTAGTGGGAT TAGTTCAAAA AACCCCAGCT CCACGGCATT
4101  AGCACTAAAT CGGAACCCTA AAGGGAGCCC CCGATTTAGA GCTTGACGGG GAAAGCCGGC GAACGTGGCG AGAAAGGAAG GGAAGAAAGC GAAAGGAGCG
      TCGTGATTTA GCCTTGGGAT TTCCCTCGGG GGCTAAATCT CGAACTGCCC CTTTCGGCCG CTTGCACCGC TCTTTCCTTC CCTTCTTTCG CTTTCCTCGC
4201  GGCGCTAGGG CGCTGGCAAG TGTAGCGGTC ACGCTGCGCG TAACCACCAC ACCCGCCGCG CTTAATGCGC CGCTACAGGG CGCGTCCCAT TCGCCATTCA
      CCGCGATCCC GCGACCGTTC ACATCGCCAG TGCGACGCGC ATTGGTGGTG TGGGCGGCGC GAATTACGCG GCGATGTCCC GCGCAGGGTA AGCGGTAAGT
4301  GGCTGCGCAA CTGTTGGGAA GGGCGATCGG TGCGGGCCTC TTCGCTATTA CGCCAGCTGG CGAAAGGGGG ATGTGCTGCA AGGCGATTAA GTTGGGTAAC
      CCGACGCGTT GACAACCCTT CCCGCTAGCC ACGCCCGGAG AAGCGATAAT GCGGTCGACC GCTTTCCCCC TACACGACGT TCCGCTAATT CAACCCATTG
                                                                                      GPD promoter
4401  GCCAGGGTTT TCCCAGTCAC GACGTTGTAA AACGACGGCC AGTGAATTGT AATACGACTC ACTATAGGGC GAATTGGCCG GTGACTCTTT CTGGCATGCG
      CGGTCCCAAA AGGGTCAGTG CTGCAACATT TTGCTGCCGG TCACTTAACA TTATGCTGAG TGATATCCCG CTTAACCGGC CACTGAGAAA GACCGTACGC
                                                 GPD promoter
4501  GAGAGACGGA CGGACGCAGA GAGAAGGGCT GAGTAATAAG CGCCACTGCG CCAGACAGCT CTGGCGGCTC TGAGGTGCAG TGGATGATTA TTAATCCGGG
      CTCTCTGCCT GCCTGCGTCT CTCTTCCCGA CTCATTATTC GCGGTGACGC GGTCTGTCGA GACCGCCGAG ACTCCACGTC ACCTACTAAT AATTAGGCCC
                                                 GPD promoter
4601  ACCGGCCGCC CCTCCGCCCC GAAGTGGAAA GGCTGGTGTG CCCCTCGTTG ACCAAGAATC TATTGCATCA TCGGAGAATA TGGAGCTTCA TCGAATCACC
      TGGCCGGCGG GGAGGCGGGG CTTCACCTTT CCGACCACAC GGGGAGCAAC TGGTTCTTAG ATAACGTAGT AGCCTCTTAT ACCTCGAAGT AGCTTAGTGG
                                                 GPD promoter
4701  GGCAGTAAGC GAAGGAGAAT GTGAAGCCAG GGGTGTATAG CCGTCGGCGA AATAGCATGC CATTAACCTA GGTACAGAAG TCCAATTGCT TCCGATCTGG
      CCGTCATTCG CTTCCTCTTA CACTTCGGTC CCCACATATC GGCAGCCGCT TTATCGTACG GTAATTGGAT CCATGTCTTC AGGTTAACGA AGGCTAGACC
                                                 GPD promoter
4801  TAAAAGATTC ACGAGATAGT ACCTTCTCCG AAGTAGGTAG AGCGAGTACC CGGCGCGTAA GCTCCCTAAT TGGCCCATCC GGCATCTGTA GGGCGTCCAA
      ATTTTCTAAG TGCTCTATCA TGGAAGAGGC TTCATCCATC TCGCTCATGG GCCGCGCATT CGAGGGATTA ACCGGGTAGG CCGTAGACAT CCCGCAGGTT
                                                 GPD promoter
4901  ATATCGTGCC TCTCCTGCTT TGCCCGGTGT ATGAAACCGG AAAGGCCGCT CAGGAGCTGG CCAGCGGCGC AGACCGGGAA CACAAGCTGG CAGTCGACCC
      TATAGCACGG AGAGGACGAA ACGGGCCACA TACTTTGGCC TTTCCGGCGA GTCCTCGACC GGTCGCCGCG TCTGGCCCTT GTGTTCGACC GTCAGCTGGG
                                                 GPD promoter
5001  ATCCGGTGCT CTGCACTCGA CCTGCTGAGG TCCCTCAGTC CCTGGTAGGC AGCTTTGCCC CGTCTGTCCG CCCGGTGTGT CGGCGGGGTT GACAAGGTCG
      TAGGCCACGA GACGTGAGCT GGACGACTCC AGGGAGTCAG GGACCATCCG TCGAAACGGG GCAGACAGGC GGGCCACACA GCCGCCCCAA CTGTTCCAGC
                                                 GPD promoter
5101  TTGCGTCAGT CCAACATTTG TTGCCATATT TTCCTGCTCT CCCCACCAGC TGCTCTTTTC TTTTCTCTTT CTTTTCCCAT CTTCAGTATA TTCATCTTCC
      AACGCAGTCA GGTTGTAAAC AACGGTATAA AAGGACGAGA GGGGTGGTCG ACGAGAAAAG AAAAGAGAAA GAAAAGGGTA GAAGTCATAT AAGTAGAAGG
                                                 GPD promoter
5201  CATCCAAGAA CCTTTATTTC CCCTAAGTAA GTACTTTGCT ACATCCATAC TCCATCCTTC CCATCCCTTA TTCCTTTGAA CCTTTCAGTT CGAGCTTTCC
      GTAGGTTCTT GGAAATAAAG GGGATTCATT CATGAAACGA TGTAGGTATG AGGTAGGAAG GGTAGGGAAT AAGGAAACTT GGAAAGTCAA GCTCGAAAGG
                      GPD promoter                                       HSVtk
5301  CACTTCATCG CAGCTTGACT AACAGCTACC CCGCTTGAGA TCGATATGGC TTCGTACCCC TGCCATCAAC ACGCGTCTGC GTTCGACCAG GCTGCGCGTT
      GTGAAGTAGC GTCGAACTGA TTGTCGATGG GGCGAACTCT AGCTATACCG AAGCATGGGG ACGGTAGTTG TGCGCAGACG CAAGCTGGTC CGACGCGCAA
```

HSVtk

5401 CTCGCGGCCA TAGCAACCGA CGTACGGCGT TGCGCCCTCG CCGGCAGCAA GAAGCCACGG AAGTCCGCCC GGAGCAGAAA ATGCCCACGC TACTGCGGGT
GAGCGCCGGT ATCGTTGGCT GCATGCCGCA ACGCGGGAGC GGCCGTCGTT CTTCGGTGCC TTCAGGCGGG CCTCGTCTTT TACGGGTGCG ATGACGCCCA

HSVtk

5501 TTATATAGAC GGTCCCCACG GGATGGGGAA AACCACCACC ACGCAACTGC TGGTGGCCCT GGGTTCGCGC GACGATATCG TCTACGTACC CGAGCCGATG
AATATATCTG CCAGGGGTGC CCTACCCCTT TTGGTGGTGG TGCGTTGACG ACCACCGGGA CCCAAGCGCG CTGCTATAGC AGATGCATGG GCTCGGCTAC

HSVtk

5601 ACTTACTGGC GGGTGCTGGG GGCTTCCGAG ACAATCGCGA ACATCTACAC CACACAACAC CGCCTCGACC AGGGTGAGAT ATCGGCCGGG GACGCGGCGG
TGAATGACCG CCCACGACCC CCGAAGGCTC TGTTAGCGCT TGTAGATGTG GTGTGTTGTG GCGGAGCTGG TCCCACTCTA TAGCCGGCCC CTGCGCCGCC

HSVtk

5701 TGGTAATGAC AAGCGCCCAG ATAACAATGG GCATGCCTTA TGCCGTGACC GACGCCGTTC TGGCTCCTCA TATCGGGGGG GAGGCTGGGA GCTCACATGC
ACCATTACTG TTCGCGGGTC TATTGTTACC CGTACGGAAT ACGGCACTGG CTGCGGCAAG ACCGAGGAGT ATAGCCCCCC CTCCGACCCT CGAGTGTACG

HSVtk

5801 CCCGCCCCCG GCCCTCACCC TCATCTTCGA CCGCCATCCC ATCGCCGCCC TCCTGTGCTA CCCGGCCGCG CGGTACCTTA TGGGCAGCAT GACCCCCCAG
GGGCGGGGGC CGGGAGTGGG AGTAGAAGCT GGCGGTAGGG TAGCGGCGGG AGGACACGAT GGGCCGGCGC GCCATGGAAT ACCCGTCGTA CTGGGGGGTC

HSVtk

5901 GCCGTGCTGG CGTTCGTGGC CCTCATCCCG CCGACCTTGC CCGGCACCAA CATCGTGCTT GGGGCCCTTC CGGAGGACAG ACACATCGAC CGCCTGGCCA
CGGCACGACC GCAAGCACCG GGAGTAGGGC GGCTGGAACG GGCCGTGGTT GTAGCACGAA CCCCGGGAAG GCCTCCTGTC TGTGTAGCTG GCGGACCGGT

HSVtk

6001 AACGCCAGCG CCCCGGCGAG CGGCTGGACC TGGCTATGCT GGCTGCGATT CGCCGCGTTT ACGGGCTACT TGCCAATACG GTGCGGTATC TGCAGTGCGG
TTGCGGTCGC GGGGCCGCTC GCCGACCTGG ACCGATACGA CCGACGCTAA GCGGCGCAAA TGCCCGATGA ACGGTTATGC CACGCCATAG ACGTCACGCC

HSVtk

6101 CGGGTCGTGG CGGGAGGACT GGGGACAGCT TTCGGGGACG GCCGTGCCGC CCCAGGGTGC CGAGCCCCAG AGCAACGCGG GCCCACGACC CCATATCGGG
GCCCAGCACC GCCCTCCTGA CCCCTGTCGA AAGCCCCTGC CGGCACGGCG GGGTCCCACG GCTCGGGGTC TCGTTGCGCC CGGGTGCTGG GGTATAGCCC

HSVtk

6201 GACACGTTAT TTACCCTGTT TCGGGCCCCC GAGTTGCTGG CCCCCAACGG CGACCTGTAT AACGTGTTTG CCTGGGCCTT GGACGTCTTG GCCAAACGCC
CTGTGCAATA AATGGGACAA AGCCCGGGGG CTCAACGACC GGGGGTTGCC GCTGGACATA TTGCACAAAC GGACCCGGAA CCTGCAGAAC CGGTTTGCGG

HSVtk

6301 TCCGTTCCAT GCACGTCTTT ATCCTGGATT ACGACCAATC GCCCGCCGGC TGCCGGGACG CCCTGCTGCA ACTTACCTCC GGGATGGTCC AGACCCACGT
AGGCAAGGTA CGTGCAGAAA TAGGACCTAA TGCTGGTTAG CGGGCGGCCG ACGGCCCTGC GGGACGACGT TGAATGGAGG CCCTACCAGG TCTGGGTGCA

HSVtk GPD terminator

6401 CACCACCCCC GGCTCCATAC CGACGATATG CGACCTGGCG CGCACGTTTG CCCGGGAGAT GGGGGAGGCT AACTGACTCG AGCTTGACCT GATCTCCCAC
GTGGTGGGGG CCGAGGTATG GCTGCTATAC GCTGGACCGC GCGTGCAAAC GGGCCCTCTA CCCCCTCCGA TTGACTGAGC TCGAACTGGA CTAGAGGGTG

GPD terminator

6501 GTCGCACAAG GTCGATGGCA ACGCCTAGAC TGCGACCAAC ACCTTGGAAC ACGGCGTCAA CGGCGCCGTT CCCGTCGTCT CGGAGACTGC GAAGCCTGCT
CAGCGTGTTC CAGCTACCGT TGCGGATCTG ACGCTGGTTG TGGAACCTTG TGCCGCAGTT GCCGCGGCAA GGGCAGCAGA GCCTCTGACG CTTCGGACGA

GPD terminator

6601 TTTCAATAAT GAAACAAACC CATGGGGGAA TTATGGAAAG GCAATGAACC AAAACAAACT AAAAAGGGGC AGCGAAGAAA AAGTCGGTAA CGTCACAACG
AAAGTTATTA CTTTGTTTGG GTACCCCCTT AATACCTTTC CGTTACTTGG TTTTGTTTGA TTTTTCCCCG TCGCTTCTTT TTCAGCCATT GCAGTGTTGC

GPD terminator

6701 ACTATTGCGG CCATGACAAC CAAGGCTTGG GTCGGCGCCG TGGGGAGTGT GGCCTAGTGC AGTGGTAGTT AGCTACGTAT GCGACTCCTA ATAAATAACA
TGATAACGCC GGTACTGTTG GTTCCGAACC CAGCCGCGGC ACCCCTCACA CCGGATCACG TCACCATCAA TCGATGCATA CGCTGAGGAT TATTTATTGT

GPD terminator

6801 AAAAAATCAG ATTAAATGAG GACCACCTTA GTAGTACTAT AGACGAAGTC ATCGAACTAC AGGGCATCTG TGCATTTTGT TGTGAACCCG TTCAAATATC
TTTTTTAGTC TAATTTACTC CTGGTGGAAT CATCATGATA TCTGCTTCAG TAGCTTGATG TCCCGTAGAC ACGTAAAACA ACACTTGGGC AAGTTTATAG

GPD terminator

6901 AAAATCATAA ACCTGCGACT TGGCTGGATG GTCAAATTCA TCCGTGTATA CACACATTCT CGCACCTTGT GAAGCAGCCA GCCGTTGCAC GCAGTTTCAT
TTTTAGTATT TGGACGCTGA ACCGACCTAC CAGTTTAAGT AGGCACATAT GTGTGTAAGA GCGTGGAACA CTTCGTCGGT CGGCAACGTG CGTCAAAGTA

GPD terminator

7001 CAGGCTTCTG AAAAGAGGAA TTAGAAAAAA AGGTATCTGT AATTAGCAGT GCAGACCATG TAATGTAATG AATACGATCC GACAAGCTCC ATTATTGAAG
GTCCGAAGAC TTTTCTCCTT AATCTTTTTT TCCATAGACA TTAATCGTCA CGTCTGGTAC ATTACATTAC TTATGCTAGG CTGTTCGAGG TAATAACTTC

GPD terminator

7101 CATTTATCAG GGTTATTGTC TCATGAGCGG ATACATATTT GAATGTATTT AGAAAAATAA ACAAATAGGG GTTCCGCGCA CATTTCCCCG AAAAGTGCCA
GTAAATAGTC CCAATAACAG AGTACTCGCC TATGTATAAA CTTACATAAA TCTTTTTATT TGTTTATCCC CAAGGCGCGT GTAAAGGGGC TTTTCACGGT

GPD terminator TrpC promoter

7201 CCTGACGTCT AAGAAACCAT TATTATCATG ACATTAACCT ATAAAAATAG GCGTATCACG AGGCCCTTTC GTCTTCAAGA ATTGTCGACA GAAGATGATA
GGACTGCAGA TTCTTTGGTA ATAATAGTAC TGTAATTGGA TATTTTTATC CGCATAGTGC TCCGGGAAAG CAGAAGTTCT TAACAGCTGT CTTCTACTAT

TrpC promoter

7301 TTGAAGGAGC ACTTTTTGGG CTTGGCTGGA GCTAGTGGAG GTCAACAATG AATGCCTATT TTGGTTTAGT CGTCCAGGCG GTGAGCACAA AATTTGTGTC
AACTTCCTCG TGAAAAACCC GAACCGACCT CGATCACCTC CAGTTGTTAC TTACGGATAA AACCAAATCA GCAGGTCCGC CACTCGTGTT TTAAACACAG

TrpC promoter

7401 GTTTGACAAG ATGGTTCATT TAGGCAACTG GTCAGATCAG CCCCACTTGT AGCAGTAGCG GCGGCGCTCG AAGTGTGACT CTTATTAGCA GACAGGAACG
CAAACTGTTC TACCAAGTAA ATCCGTTGAC CAGTCTAGTC GGGGTGAACA TCGTCATCGC CGCCGCGAGC TTCACACTGA GAATAATCGT CTGTCCTTGC

TrpC promoter

7501 AGGACATTAT TATCATCTGC TGCTTGGTGC ACGATAACTT GGTGCGTTTG TCAAGCAAGG TAAGTGAACG ACCCGGTCAT ACCTTCTTAA GTTCGCCCTT
TCCTGTAATA ATAGTAGACG ACGAACCACG TGCTATTGAA CCACGCAAAC AGTTCGTTCC ATTCACTTGC TGGGCCAGTA TGGAAGAATT CAAGCGGGAA

TrpC promoter

7601 CCTCCCTTTA TTTCAGATTC AATCTGACTT ACCTATTCTA CCCAAGCATC GATAAGCTTG ATATCG
GGAGGGAAAT AAAGTCTAAG TTAGACTGAA TGGATAAGAT GGGTTCGTAG CTATTCGAAC TATAGC

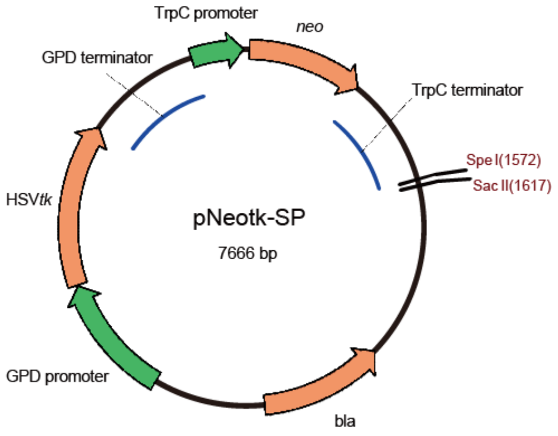

neo

1 AATTCAGAGC CCATGGGATC GGCCATTGAA CAAGATGGAT TGCACGCAGG TTCTCCGGCC GCTTGGGTGG AGAGGCTATT CGGCTATGAC TGGGCACAAC

neo

101 AGACAATCGG CTGCTCTGAT GCCGCCGTGT TCCGGCTGTC AGCGCAGGGG CGCCCGGTTC TTTTTGTCAA GACCGACCTG TCCGGTGCCC TGAATGAACT

neo

201 GCAGGACGAG GCAGCGCGGC TATCGTGGCT GGCCACGACG GGCGTTCCTT GCGCAGCTGT GCTCGACGTT GTCACTGAAG CGGGAAGGGA CTGGCTGCTA

neo

301 TTGGGCGAAG TGCCGGGGCA GGATCTCCTG TCATCTCACC TTGCTCCTGC CGAGAAAGTA TCCATCATGG CTGATGCAAT GCGGCGGCTG CATACGCTTG

neo

401 ATCCGGCTAC CTGCCCATTC GACCACCAAG CGAAACATCG CATCGAGCGA GCACGTACTC GGATGGAAGC CGGTCTTGTC GATCAGGATG ATCTGGACGA

neo

501 AGAGCATCAG GGGCTCGCGC CAGCCGAACT GTTCGCCAGG CTCAAGGCGC GCATGCCCGA CGGCGAGGAT CTCGTCGTGA CCCATGGCGA TGCCTGCTTG

neo

601 CCGAATATCA TGGTGGAAAA TGGCCGCTTT TCTGGATTCA TCGACTGTGG CCGGCTGGGT GTGGCGGACC GCTATCAGGA CATAGCGTTG GCTACCCGTG

neo

701 ATATTGCTGA AGAGCTTGGC GGCGAATGGG CTGACCGCTT CCTCGTGCTT TACGGTATCG CCGCTCCCGA TTCGCAGCGC ATCGCCTTCT ATCGCCTTCT

neo TrpC terminator

801 TGACGAGTTC TTCTGACGGG CTGGCTGCAG TGGTGGGGGA TCCACTTAAC GTTACTGAAA TCATCAAACA GCTTGACGAA TCTGGATATA AGATCGTTGG

TrpC terminator

901 TGTCGATGTC AGCTCCGGAG TTGAGACAAA TGGTGTTCAG GATCTCGATA AGATACGTTC ATTTGTCCAA GCAGCAAAGA GTGCCTTCTA GTGATTTAAT

TrpC terminator

1001 AGCTCCATGT CAACAAGAAT AAAACGCGTT TCGGGTTTAC CTCTTCCAGA TACAGCTCAT CTGCAATGCA TTAATGCATT GGACCTCGCA ACCCTAGTAC

TrpC terminator

1101 GCCCTTCAGG CTCCGGCGAA GCAGAAGAAT AGCTTAGCAG AGTCTATTTT CATTTTCGGG AGACGAGATC AAGCAGATCA ACGGTCGTCA AGAGACCTAC

TrpC terminator

1201 GAGACTGAGG AATCCGCTCT TGGCTCCACG CGACTATATA TTTGTCTCTA ATTGTACTTT GACATGCTCC TCTTCTTTAC TCTGATAGCT TGACTATGAA

TrpC terminator

1301 AATTCCGTCA CCAGCCCCTG GGTTCGCAAA GATAATTGCA CTGTTTCTTC CTTGAACTCT CAAGCCTACA GGACACACAT TCATCGTAGG TATAAACCTC

TrpC terminator

1401 GAAAATCATT CCTACTAAGA TGGGTATACA ATAGTAACCA TGGTTGCCTA GTGAATGCTC CGTAACACCC AATACGCCGG CCGAAACTTT TTTACAACTC

Tri10 upstream region

TrpC terminator DjTri10inv_speI_AS

SpeI

1501 TCCTATGAGT CGTTTACCCA GAATGCACAG GTACACTTGT TTAGAGGTAA TCCTTCTTTC TAGAGGATCC ACTAGTATCC ATGATGACTA ACGACAATAA

Tri10 upstream region

1601 TAATGATAAT TTGATGATTG TTGGTTGGCA TACAATTGAA GTTGGGGGAA AGACGGGAAG GAATGGGCGT GGAGAAGTTA AACGATGTTA CAATATAATG

Tri10 upstream region

1701 CAAGGATAGA GTTTTACAGA AGTGTCGTGT CCCTCATAGT GCTCAAGCAG GCATCAAACC TTATCTTGTG TGGTTTCTGT ACGCCTCGTT CAGTGGACGT

Tri10 upstream region

1801 TTGGGTGGTG CGAAAAGGCA AGGGTATGGT CACTGATCTG GACAAAACGC AAGCGCTTTG AAGATCAATT CGCGGGTCTA GAGAATGTGC CAGCTCCCTC

Tri10 upstream region

1901 CAACCTCCGA GCGGGTACAA GATTCCCGCA TCGCACTACA GTTCGTAGCG CCAGTCTACC AGTCTGCCAG CTGCAGCTGC AGATGCAGTT GCAGGGTTGG

Tri10 upstream region

2001 GGGTAGCCCT GTCGCACCGA CGGGTGTACA GACTTGGTTA CGCAAAAGCC TGCACTCAAG TGGGAAATGG TTACAGGCTC AGGTTACGTA CAGACATTCA

Tri10 upstream region

2101 GTTTCAACTG CCTATTCCGA AGTAATTATC CTTGTTCAAC TGTTCATTCT AACAAAACAC GACCGAACTA TTTGTAATCA TTATAACAGA CCACCATTCT

Tri10 upstream region

Tri10 downstream region

DjTri10_NheI_AS

NheI

2201 CCAAACGAGT ATCCGTACCC GCAAAACATA CTTCCAAACT CGCCTGCTAG CCACTCCGTC ACAACATAGA TATGCTTTCA TTAATTTTCG CTTTTCGCGC

DjTri10_NheI_S

Tri10 downstream region

2301 TGTTGCTGTA GGGCAGCAAA TGCCATGCAT TAAATTAAAA AGAAGGGACC TATTCAAGGG TTCACACACA CCGACGGCAG ATTTTACGTC TCTGCCGGCC

Tri10 downstream region

2401 TGTCATGAAA GCTTAAAAAG AACAGACCCT TGGATGTAAC CCACCATCAG TTGAACATGG GTTTGTCGGC GCCGACAAGT CCGTTGCCAT GTTGCCGACG

Tri10 downstream region

2501 AGAGAAGAGT GATCGTGAAG CTCGATGGCA TTCTATGCTG AGCCTCAATG GCGATGGAAT CGGTAATGGC AATGCTAGAC GAGACGCCGT TTAGGCCGGA

Tri10 downstream region

2601 TGTTCGGTTC GACGCTTGCA TATTCAGGCC TTTTACTGAC GTTTCAGCTT CACACCAACT CAGCATCATT CAGGCCTTTT TCTCGCATAT CTGCGATATT

Tri10 downstream region

2701 TTTGGTCGGT AGATAGAGCA TAGACATTAA GACAATTAAG ATATTAACCA TCGGCCAGAA CCGATTAGAA ACATCTCAAC AATTTCTTCC CATCAAACAA

Tri10 downstream region

2801 ACACGAACAC GACATCAATT GCATCACACC ACTACCACTA CTACTACTAC TATCCACTCA AACACTCACC CCCAATCTCC AAGATGCTCG CAGCCGCTAA

Tri10 downstream region

2901 ACTGATCGAC TCATATGAGA TGGACCCTGA TGTCTCGTGG CTCGAGGTTT TCGCATACTC GGGAGTTAGC GCTGCTTTAT GCGCTACCAT ATGGGTCGCA

Tri10 downstream region

3001 GCCAAAGCAT GCTGAGACTG CAGTGCCAGA TCAGCACCTC TATCATCTAC CCAGCCCAAC AGAGGAAGAA TTGCCTGTCG CAAATCTCAG CATTCTTCCA

Tri10 downstream region

3101 CTTCGGCCCC ACGAATGAAT CATTTCCCAA CTGGGTTTCG TTGCGGGGAT CTTCACAAGG AACTTCGACA AATCCCCTCG CTTGAAATCA ACACTCCTTC

Tri10 downstream region

3201 ACTATGATCA ATATCGTTTG TCGTACGGGC TAAACAATGT CAAGCCATGT CGAGACAATC AAGGGACACA GCACTGATTC GCCCACCATG GTTCCAGACT

Tri10 downstream region

3301 GCTTTCAGCA CGATTGGTTG GATAGATGGA GAAGATGCGG TTCACGGTTA AAAAAGTATA CTAGAAATAT ATATTGAAAG TCCATCATTC AAGTTACATG

Tri10 downstream region

3401 GGAAACGTTT TCCGCTCTTC TATTCCATTT AAATCATGCA TCATGCCAAT CCCTGTTTAA ACGCCAAAAT GAGTCGAACC CATAACATCT AATAAAACTT

Tri10 downstream region

3501 CCCTGCATGC ATCATCTGTA AGTGAGCATC AAACTTACAT GTATCGGGTA GCTGATGCAG AAGAAAGACT TCGGGTATCG GTATCCGTAG TCTCTGCGCC

DjTri10inv_SacII_S

Tri10 downstream re

SacII

3601 GATACCATCC GCGGTGGAGC TCCAGCTTTT GTTCCCTTTA GTGAGGGTTA ATTTCGAGCT TGGCGTAATC ATGGTCATAG CTGTTTCCTG TGTGAAATTG

DjTri10inv_SacII_S

3701 TTATCCGCTC ACAATTCCAC ACAACATACG AGCCGGAAGC ATAAAGTGTA AAGCCTGGGG TGCCTAATGA GTGAGCTAAC TCACATTAAT TGCGTTGCGC

3801 TCACTGCCCG CTTTCCAGTC GGGAAACCTG TCGTGCCAGC TGCATTAATG AATCGGCCAA CGCGCGGGGA GAGGCGGTTT GCGTATTGGG CGCTCTTCCG

3901 CTTCCTCGCT CACTGACTCG CTGCGCTCGG TCGTTCGGCT GCGGCGAGCG GTATCAGCTC ACTCAAAGGC GGTAATACGG TTATCCACAG AATCAGGGGA

4001 TAACGCAGGA AAGAACATGT GAGCAAAAGG CCAGCAAAAG GCCAGGAACC GTAAAAAGGC CGCGTTGCTG GCGTTTTTCC ATAGGCTCCG CCCCCCTGAC

4101 GAGCATCACA AAAATCGACG CTCAAGTCAG AGGTGGCGAA ACCCGACAGG ACTATAAAGA TACCAGGCGT TTCCCCCTGG AAGCTCCCTC GTGCGCTCTC

4201 CTGTTCCGAC CCTGCCGCTT ACCGGATACC TGTCCGCCTT TCTCCCTTCG GGAAGCGTGG CGCTTTCTCA TAGCTCACGC TGTAGGTATC TCAGTTCGGT

4301 GTAGGTCGTT CGCTCCAAGC TGGGCTGTGT GCACGAACCC CCCGTTCAGC CCGACCGCTG CGCCTTATCC GGTAACTATC GTCTTGAGTC CAACCCGGTA

4401 AGACACGACT TATCGCCACT GGCAGCAGCC ACTGGTAACA GGATTAGCAG AGCGAGGTAT GTAGGCGGTG CTACAGAGTT CTTGAAGTGG TGGCCTAACT

4501 ACGGCTACAC TAGAAGGACA GTATTTGGTA TCTGCGCTCT GCTGAAGCCA GTTACCTTCG GAAAAAGAGT TGGTAGCTCT TGATCCGGCA AACAAACCAC

4601 CGCTGGTAGC GGTGGTTTTT TTGTTTGCAA GCAGCAGATT ACGCGCAGAA AAAAAGGATC TCAAGAAGAT CCTTTGATCT TTTCTACGGG GTCTGACGCT

4701 CAGTGGAACG AAAACTCACG TTAAGGGATT TTGGTCATGA GATTATCAAA AAGGATCTTC ACCTAGATCC TTTTAAATTA AAAATGAAGT TTTAAATCAA

4801 TCTAAAGTAT ATATGAGTAA ACTTGGTCTG ACAGTTACCA ATGCTTAATC AGTGAGGCAC CTATCTCAGC GATCTGTCTA TTTCGTTCAT CCATAGTTGC

bla

4901 CTGACTCCCC GTCGTGTAGA TAACTACGAT ACGGGAGGGC TTACCATCTG GCCCCAGTGC TGCAATGATA CCGCGAGACC CACGCTCACC GGCTCCAGAT

bla

5001 TTATCAGCAA TAAACCAGCC AGCCGGAAGG GCCGAGCGCA GAAGTGGTCC TGCAACTTTA TCCGCCTCCA TCCAGTCTAT TAATTGTTGC CGGGAAGCTA

bl[illegible]

5101 GAGTAAGTAG TTCGCCAGTT AATAGTTTGC GCAACGTTGT TGCCATTGCT ACAGGCATCG TGGTGTCACG CTCGTCGTTT GGTATGGCTT CATTCAGCTC

bla

5201 CGGTTCCCAA CGATCAAGGC GAGTTACATG ATCCCCCATG TTGTGCAAAA AAGCGGTTAG CTCCTTCGGT CCTCCGATCG TTGTCAGAAG TAAGTTGGCC

bla

5301 GCAGTGTTAT CACTCATGGT TATGGCAGCA CTGCATAATT CTCTTACTGT CATGCCATCC GTAAGATGCT TTTCTGTGAC TGGTGAGTAC TCAACCAAGT

bla

5401 CATTCTGAGA ATAGTGTATG CGGCGACCGA GTTGCTCTTG CCCGGCGTCA ATACGGGATA ATACCGCGCC ACATAGCAGA ACTTTAAAAG TGCTCATCAT

bla

5501 TGGAAAACGT TCTTCGGGGC GAAAACTCTC AAGGATCTTA CCGCTGTTGA GATCCAGTTC GATGTAACCC ACTCGTGCAC CCAACTGATC TTCAGCATCT

bla

5601 TTTACTTTCA CCAGCGTTTC TGGGTGAGCA AAAACAGGAA GGCAAAATGC CGCAAAAAAG GGAATAAGGG CGACACGGAA ATGTTGAATA CTCATACTCT

bla

5701 TCCTTTTTCA ATATTATTGA AGCATTTATC AGGGTTATTG TCTCATGAGC GGATACATAT TTGAATGTAT TTAGAAAAAT AAACAAATAG GGGTTCCGCG

5801 CACATTTCCC CGAAAAGTGC CACCTAAATT GTAAGCGTTA ATATTTTGTT AAAATTCGCG TTAAATTTTT GTTAAATCAG CTCATTTTTT AACCAATAGG

5901 CCGAAATCGG CAAAATCCCT TATAAATCAA AAGAATAGAC CGAGATAGGG TTGAGTGTTG TTCCAGTTTG GAACAAGAGT CCACTATTAA AGAACGTGGA

6001 CTCCAACGTC AAAGGGCGAA AAACCGTCTA TCAGGGCGAT GGCCCACTAC GTGAACCATC ACCCTAATCA AGTTTTTTGG GGTCGAGGTG CCGTAAAGCA

6101 CTAAATCGGA ACCCTAAAGG GAGCCCCCGA TTTAGAGCTT GACGGGGAAA GCCGGCGAAC GTGGCGAGAA AGGAAGGGAA GAAAGCGAAA GGAGCGGGCG

6201 CTAGGGCGCT GGCAAGTGTA GCGGTCACGC TGCGCGTAAC CACCACACCC GCCGCGCTTA ATGCGCCGCT ACAGGGCGCG TCCCATTCGC CATTCAGGCT

6301 GCGCAACTGT TGGGAAGGGC GATCGGTGCG GGCCTCTTCG CTATTACGCC AGCTGGCGAA AGGGGGATGT GCTGCAAGGC GATTAAGTTG GGTAACGCCA

GPD promoter

6401 GGGTTTTCCC AGTCACGACG TTGTAAAACG ACGGCCAGTG AATTGTAATA CGACTCACTA TAGGGCGAAT TGGCCGGTGA CTCTTTCTGG CATGCGGAGA

GPD promoter

6501 GACGGACGGA CGCAGAGAGA AGGGCTGAGT AATAAGCGCC ACTGCGCCAG ACAGCTCTGG CGGCTCTGAG GTGCAGTGGA TGATTATTAA TCCGGGACCG

GPD promoter

6601 GCCGCCCCTC CGCCCCGAAG TGGAAAGGCT GGTGTGCCCC TCGTTGACCA AGAATCTATT GCATCATCGG AGAATATGGA GCTTCATCGA ATCACCGGCA

GPD promoter

6701 GTAAGCGAAG GAGAATGTGA AGCCAGGGGT GTATAGCCGT CGGCGAAATA GCATGCCATT AACCTAGGTA CAGAAGTCCA ATTGCTTCCG ATCTGGTAAA

GPD promoter

6801 AGATTCACGA GATAGTACCT TCTCCGAAGT AGGTAGAGCG AGTACCCGGC GCGTAAGCTC CCTAATTGGC CCATCCGGCA TCTGTAGGGC GTCCAAATAT

GPD promoter

6901 CGTGCCTCTC CTGCTTTGCC CGGTGTATGA AACCGGAAAG GCCGCTCAGG AGCTGGCCAG CGGCGCAGAC CGGGAACACA AGCTGGCAGT CGACCCATCC

GPD promoter

7001 GGTGCTCTGC ACTCGACCTG CTGAGGTCCC TCAGTCCCTG GTAGGCAGCT TTGCCCCGTC TGTCCGCCCG GTGTGTCGGC GGGGTTGACA AGGTCGTTGC

GPD promoter

7101 GTCAGTCCAA CATTTGTTGC CATATTTTCC TGCTCTCCCC ACCAGCTGCT CTTTTCTTTT CTCTTTCTTT TCCCATCTTC AGTATATTCA TCTTCCCATC

GPD promoter

7201 CAAGAACCTT TATTTCCCCT AAGTAAGTAC TTTGCTACAT CCATACTCCA TCCTTCCCAT CCCTTATTCC TTTGAACCTT TCAGTTCGAG CTTTCCCACT

GPD promoter HSVtk

7301 TCATCGCAGC TTGACTAACA GCTACCCCGC TTGAGATCGA TATGGCTTCG TACCCCTGCC ATCAACACGC GTCTGCGTTC GACCAGGCTG CGCGTTCTCG

HSVtk

7401 CGGCCATAGC AACCGACGTA CGGCGTTGCG CCCTCGCCGG CAGCAAGAAG CCACGGAAGT CCGCCCGGAG CAGAAAATGC CCACGCTACT GCGGGTTTAT

HSVtk

7501 ATAGACGGTC CCCACGGGAT GGGGAAAACC ACCACCACGC AACTGCTGGT GGCCCTGGGT TCGCGCGACG ATATCGTCTA CGTACCCGAG CCGATGACTT

HSVtk

7601 ACTGGCGGGT GCTGGGGGCT TCCGAGACAA TCGCGAACAT CTACACCACA CAACACCGCC TCGACCAGGG TGAGATATCG GCCGGGGACG CGGCGGTGGT

HSVtk

7701 AATGACAAGC GCCCAGATAA CAATGGGCAT GCCTTATGCC GTGACCGACG CCGTTCTGGC TCCTCATATC GGGGGGGAGG CTGGGAGCTC ACATGCCCCG

HSVtk

7801 CCCCCGGCCC TCACCCTCAT CTTCGACCGC CATCCCATCG CCGCCCTCCT GTGCTACCCG GCCGCGCGGT ACCTTATGGG CAGCATGACC CCCCAGGCCG

HSVtk

7901 TGCTGGCGTT CGTGGCCCTC ATCCCGCCGA CCTTGCCCGG CACCAACATC GTGCTTGGGG CCCTTCCGGA GGACAGACAC ATCGACCGCC TGGCCAAACG

HSVtk

8001 CCAGCGCCCC GGCGAGCGGC TGGACCTGGC TATGCTGGCT GCGATTCGCC GCGTTTACGG GCTACTTGCC AATACGGTGC GGTATCTGCA GTGCGGCGGG

HSVtk

8101 TCGTGGCGGG AGGACTGGGG ACAGCTTTCG GGGACGGCCG TGCCGCCCCA GGGTGCCGAG CCCCAGAGCA ACGCGGGCCC ACGACCCCAT ATCGGGGACA

HSVtk

8201 CGTTATTTAC CCTGTTTCGG GCCCCCGAGT TGCTGGCCCC CAACGGCGAC CTGTATAACG TGTTTGCCTG GGCCTTGGAC GTCTTGGCCA AACGCCTCCG

HSVtk

8301 TTCCATGCAC GTCTTTATCC TGGATTACGA CCAATCGCCC GCCGGCTGCC GGGACGCCCT GCTGCAACTT ACCTCCGGGA TGGTCCAGAC CCACGTCACC

HSVtk GPD terminator

8401 ACCCCCGGCT CCATACCGAC GATATGCGAC CTGGCGCGCA CGTTTGCCCG GGAGATGGGG GAGGCTAACT GACTCGAGCT TGACCTGATC TCCCACGTCG

GPD terminator
8501 CACAAGGTCG ATGGCAACGC CTAGACTGCG ACCAACACCT TGGAACACGG CGTCAACGGC GCCGTTCCCG TCGTCTCGGA GACTGCGAAG CCTGCTTTTC

GPD terminator
8601 AATAATGAAA CAAACCCATG GGGGAATTAT GGAAAGGCAA TGAACCAAAA CAAACTAAAA AGGGGCAGCG AAGAAAAAGT CGGTAACGTC ACAACGACTA

GPD terminator
8701 TTGCGGCCAT GACAACCAAG GCTTGGGTCG GCGCCGTGGG GAGTGTGGCC TAGTGCAGTG GTAGTTAGCT ACGTATGCGA CTCCTAATAA ATAACAAAAA

GPD terminator
8801 AATCAGATTA AATGAGGACC ACCTTAGTAG TACTATAGAC GAAGTCATCG AACTACAGGG CATCTGTGCA TTTTGTTGTG AACCCGTTCA AATATCAAAA

GPD terminator
8901 TCATAAACCT GCGACTTGGC TGGATGGTCA AATTCATCCG TGTATACACA CATTCTCGCA CCTTGTGAAG CAGCCAGCCG TTGCACGCAG TTTCATCAGG

GPD terminator
9001 CTTCTGAAAA GAGGAATTAG AAAAAAAGGT ATCTGTAATT AGCAGTGCAG ACCATGTAAT GTAATGAATA CGATCCGACA AGCTCCATTA TTGAAGCATT

GPD terminator
9101 TATCAGGGTT ATTGTCTCAT GAGCGGATAC ATATTTGAAT GTATTTAGAA AAATAAACAA ATAGGGGTTC CGCGCACATT TCCCCGAAAA GTGCCACCTG

GPD terminator TrpC promoter
9201 ACGTCTAAGA AACCATTATT ATCATGACAT TAACCTATAA AAATAGGCGT ATCACGAGGC CCTTTCGTCT TCAAGAATTG TCGACAGAAG ATGATATTGA

TrpC promoter
9301 AGGAGCACTT TTTGGGCTTG GCTGGAGCTA GTGGAGGTCA ACAATGAATG CCTATTTTGG TTTAGTCGTC CAGGCGGTGA GCACAAAATT TGTGTCGTTT

TrpC promoter
9401 GACAAGATGG TTCATTTAGG CAACTGGTCA GATCAGCCCC ACTTGTAGCA GTAGCGGCGG CGCTCGAAGT GTGACTCTTA TTAGCAGACA GGAACGAGGA

TrpC promoter
9501 CATTATTATC ATCTGCTGCT TGGTGCACGA TAACTTGGTG CGTTTGTCAA GCAAGGTAAG TGAACGACCC GGTCATACCT TCTTAAGTTC GCCCTTCCTC

TrpC promoter
9601 CCTTTATTTC AGATTCAATC TGACTTACCT ATTCTACCCA AGCATCGATA AGCTTGATAT CG

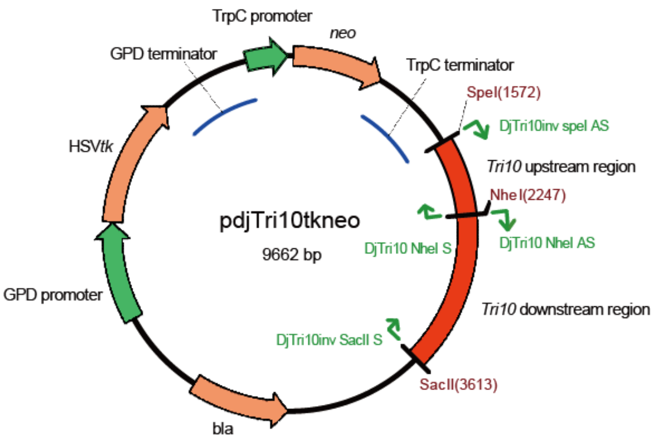

1 TCGCGCGTTT CGGTGATGAC GGTGAAAACC TCTGACACAT GCAGCTCCCG GAGACGGTCA CAGCTTGTCT GTAAGCGGAT GCCGGGAGCA GACAAGCCCG
101 TCAGGGCGCG TCAGCGGGTG TTGGCGGGTG TCGGGGCTGG CTTAACTATG CGGCATCAGA GCAGATTGTA CTGAGAGTGC ACCATATGCG GTGTGAAATA
201 CCGCACAGAT GCGTAAGGAG AAAATACCGC ATCAGGCGCC ATTCGCCATT CAGGCTGCGC AACTGTTGGG AAGGGCGATC GGTGCGGGCC TCTTCGCTAT

CjT10pro_S
301 TACGCCAGCT GGCGAAAGGG GGATGTGCTG CAAGGCGATT AAGTTGGGTA ACGCCAGGGT TTTCCCAGTC ACGACGTTGT AAAACGACGG CCAGTGAATT

CjT10pro_S
Tri10 upstream region
401 CGAGCTCGGT ACCCAGGCGA GTTTGGAAGT ATGTTTTGCG GGTACGGATA CTCGTTTGGA GAATGGTGGT CTGTTATAAT GATTACAAAT AGTTCGGTCG

Tri10 upstream region
501 TGTTTTGTTA GAATGAACAG TTGAACAAGG ATAATTACTT CGGAATAGGC AGTTGAAACT GAATGTCTGT ACGTAACCTG AGCCTGTAAC CATTTCCCAC

Tri10 upstream region
601 TTGAGTGCAG GCTTTTGCGT AACCAAGTCT GTACACCCGT CGGTGCGACA GGGCTACCCC CAACCCTGCA ACTGCATCTG CAGCTGCAGC TGGCAGACTG

Tri10 upstream region
701 GTAGACTGGC GCTACGAACT GTAGTGCGAT GCGGGAATCT TGTACCCGCT CGGAGGTTGG AGGGAGCTGG CACATTCTCT AGACCCGCGA ATTGATCTTC

Tri10 upstream region
801 AAAGCGCTTG CGTTTTGTCC AGATCAGTGA CCATACCCTT GCCTTTTCGC ACCACCCAAA CGTCCACTGA ACGAGGCGTA CAGAAACCAC ACAAGATAAG

Tri10 upstream region

901 GTTTGATGCC TGCTTGAGCA CTATGAGGGA CACGACACTT CTGTAAAACT CTATCCTTGC ATTATATTGT AACATCGTTT AACTTCTCCA CGCCCATTCC

Tri10nsm

Tri10 upstream region

+1 M D F P K P R Q ·

1001 TTCCCGTCTT TCCCCCAACT TCAATTGTAT GCCAACCAAC AATCATCAAA TTATCATTAT TATTGTCGTT AGTCATCATG GATTTCCCAA AGCCTAGACA

Tri10nsm

+1 Q V R E T S L L M Y Y L D V V F P L Q C I N P N N N C L G K R E W L

1101 AGTCCGAGAG ACGAGCCTGT TGATGTACTA CCTAGACGTC GTGTTTCCTC TGCAATGCAT CAACCCAAAC AACAATTGTC TGGGAAAGAG AGAGTGGCTG

Tri10nsm

CjT10nsm1_S

+1 L T I L T S A R P T Y Y A T L C M S L L Y K E S L S S P C R S E Q A ·

1201 TTGACTATAC TGACCTCTGC GCGGCCTACG TACTATGCCA CATTGTGCAT GTCGCTCCTC TATAAAGAAT CGCTTTCAAG CCCTTGCAGA TCTGAACAGG

CjT10nsm1_AS

Tri10nsm

CjT10nsm1_S

+1 · A *

1301 [illegible]GTAGGTATG GAAGAGAGAG AAGACATACT ACTACATTCT TGCACTCCAG GAGTCTCAGA AGCTGCTGGG TGGGCTCGAC AAGACATTTG GCATCACAAG

nonsense mutation

CjT10nsm1_AS

Tri10nsm

1401 GCTGAAAGGT ACCGTCGTTG CCCTTGCTTG CATGCTACAG CTTATCAGTT TTGAGGTAAG ACGAATCCAC CATTGTTTCG ATGCTCGATG TCGATGCTCG

Tri10nsm

1501 ATATCCGATC TACGATTATC GTTGGTCACT AACAAATTAA AATAGTCTTC GCACCTAAGC AGGGGAGATT GGCGCGTTCA CCTCCATGCG GCCAACATAC

Tri10nsm

1601 TCATTCCTGT CTTGGTTGAG GGATGGTCCA CAGCTTTGCA ATCAGGCCCC CCAGCCACCT CCATATGGTG CGAGCTGGAT GAATCACACT TCGGCTCGAC

Tri10nsm

1701 TGAAGATCAA ACCTCTTTGA GCTTCGAATA CGTCGGAGCT TTGAGATTCC TGTCAAACTC ACTCGCCGCA GTCGGCATCC TGTCTTGCAT ATCTATTGGC

Tri10nsm

1801 CCATCAGCAC CATTTGAAGA TTACGGCCAT CTCCTGGACC AGCCAGGCCT TATACAGCTG GACGAGGTGC TGGGGTGCAG GAATTGGACC ATGTTGACTA

Tri10nsm

1901 TTCTCGAAGT GGGTAAGCTG GATCGTTGGA AGCGACAGGA GCAAGAACAT AATCGCTTGA GCCTAAAGAC GCTCGCTAGG CGCGCCATGA TGATTGAGGA

Tri10nsm

2001 TATGTTGTCA GACGAGCTAC AAAGGCTACC GACAGACGAG ACGCTTCCAG ACCTCATCAC TCAGATTTAC GCCGCCTCTA TCATGACGTA TCTGCATACA

Tri10nsm

2101 GTAGTTTCCG GACTCAATCC CAACCTTTCA GAGGTTCAGG ATAGTGTGGC CGGGACGCTT CAATTGTTGG AGAGGCTCCC AAATCTTGAA GCTGTCACGA

Tri10nsm

2201 GCGTTACTTG GCCTCTAGCT GTCACAGGAT GCATGGCCTC AGAAAGTCAT AAGGACTTTT TCAGAAATAC TCTGAGGTCG TATGAGGCGA CATTCAGCTC

Tri10nsm

2301 CTTAAAAAAG TATGACGGAA CTCTTCAGGT CTTGGAAGAC GCTTGGAAGA GAAGAGAGAT AGATACAGAG TCTCCAATGA GATGGGAAGA CTTGACGGAT

Tri10nsm

Tri10_downstream region

2401 CACCATGGGC TTCCAGTGCT ACTTTGGTAG GGATGGTATC GGCGCAGAGA CTACGGATAC CGATACCCGA AGTCTTTCTT CTGCATCAGC TACCCGATAC

Tri10_downstream region

2501 ATGTAAGTTT GATGCTCACT TACAGATGAT GCATGCAGGG AAGTTTTATT AGATGTTATG GGTTCGACTC ATTTTGGCGT TTAAACAGGG ATTGGCATGA

Tri10_downstream region

2601 TGCATGATTT AAATGGAATA GAAGAGCGGA AAACGTTTCC CATGTAACTT GAATGATGGA CTTTCAATAT ATATTTCTAG TATACTTTTT TAACCGTGAA

Tri10_downstream region

2701 CCGCATCTTC TCCATCTATC CAACCAATCG TGCTGAAAGC AGTCTGGAAC CATGGTGGGC GAATCAGTGC TGTGTCCCTT GATTGTCTCG ACATGGCTTG

Tri10_downstream region

2801 ACATTGTTTA GCCCGTACGA CAAACGATAT TGATCATAGT GAAGGAGTGT TGATTTCAAG CGAGGGGATT TGTCGAAGTT CCTTGTGAAG ATCCCCGCAA

Tri10_downstream region

2901 CGAAACCCAG TTGGGAAATG ATTCATTCGT GGGGCCGAAG TGGAAGAATG CTGAGATTTG CGACAGGCAA TTCTTCCTCT GTTGGGCTGG GTAGATGATA

Tri10_downstream region

3001 GAGGTGCTGA TCTGGCACTG CAGTCTCAGC ATGCTTTGGC TGCGACCCAT ATGGTAGCGC ATAAAGCAGC GCTAACTCCC GAGTATGCGA AAACCTCGAG

Tri10_downstream region

3101 CCACGAGACA TCAGGGTCCA TCTCATATGA GTCGATCAGT TTAGCGGCTG CGAGCATCTT GGAGATTGGG GGTGAGTGTT TGAGTGGATA GTAGTAGTAG

Tri10_downstream region

3201 TAGTGGTAGT GGTGTGATGC AATTGATGTC GTGTTCGTGT TTGTTTGATG GGAAGAAATT GTTGAGATGT TTCTAATCGG TTCTGGCCGA TGGTTAATAT

Tri10_downstream region
3301 CTTAATTGTC TTAATGTCTA TGCTCTATCT ACCGACCAAA AATATCGCAG ATATGCGAGA AAAAGGCCTG AATGATGCTG AGTTGGTGTG AAGCTGAAAC

Tri10_downstream region
3401 GTCAGTAAAA GGCCTGAATA TGCAAGCGTC GAACCGAACA TCCGGCCTAA ACGGCGTCTC GTCTAGCATT GCCATTACCG ATTCCATCGC CATTGAGGCT

Tri10_downstream region
3501 CAGCATAGAA TGCCATCGAG CTTCACGATC ACTCTTCTCT CGTCGGCAAC ATGGCAACGG ACTTGTCGGC GCCGACAAAC CCATGTTCAA CTGATGGTGG

Tri10_downstream region
3601 GTTACATCCA AGGGTCTGTT CTTTTTAAGC TTTCATGACA GGCCGGCAGA GACGTAAAAT CTGCCGTCGG TGTGTGTGAA CCCTTGAATA GGTCCCTTCT

Tri10_downstream region BamHI
3701 TTTTAATTTA ATGCATGGCA TTTGCTGCCC TACAGCAACA GCGCGAAAAG CGAAAATTAA TGAAAGCATA TCTATGTTGT GACGGAGTGG GGGATCCTCT
CjT10ter_AS

3801 AGAGTCGACC TGCAGGCATG CAAGCTTGGC GTAATCATGG TCATAGCTGT TTCCTGTGTG AAATTGTTAT CCGCTCACAA TTCCACACAA CATACGAGCC
CjT10ter_AS

3901 GGAAGCATAA AGTGTAAAGC CTGGGGTGCC TAATGAGTGA GCTAACTCAC ATTAATTGCG TTGCGCTCAC TGCCCGCTTT CCAGTCGGGA AACCTGTCGT
4001 GCCAGCTGCA TTAATGAATC GGCCAACGCG CGGGGAGAGG CGGTTTGCGT ATTGGGCGCT CTTCCGCTTC CTCGCTCACT GACTCGCTGC GCTCGGTCGT
4101 TCGGCTGCGG CGAGCGGTAT CAGCTCACTC AAAGGCGGTA ATACGGTTAT CCACAGAATC AGGGGATAAC GCAGGAAAGA ACATGTGAGC AAAAGGCCAG
4201 CAAAAGGCCA GGAACCGTAA AAAGGCCGCG TTGCTGGCGT TTTTCCATAG GCTCCGCCCC CCTGACGAGC ATCACAAAAA TCGACGCTCA AGTCAGAGGT
4301 GGCGAAACCC GACAGGACTA TAAAGATACC AGGCGTTTCC CCCTGGAAGC TCCCTCGTGC GCTCTCCTGT TCCGACCCTG CCGCTTACCG GATACCTGTC
4401 CGCCTTTCTC CCTTCGGGAA GCGTGGCGCT TTCTCAATGC TCACGCTGTA GGTATCTCAG TTCGGTGTAG GTCGTTCGCT CCAAGCTGGG CTGTGTGCAC
4501 GAACCCCCCG TTCAGCCCGA CCGCTGCGCC TTATCCGGTA ACTATCGTCT TGAGTCCAAC CCGGTAAGAC ACGACTTATC GCCACTGGCA GCAGCCACTG
4601 GTAACAGGAT TAGCAGAGCG AGGTATGTAG GCGGTGCTAC AGAGTTCTTG AAGTGGTGGC CTAACTACGG CTACACTAGA AGGACAGTAT TTGGTATCTG
4701 CGCTCTGCTG AAGCCAGTTA CCTTCGGAAA AAGAGTTGGT AGCTCTTGAT CCGGCAAACA AACCACCGCT GGTAGCGGTG GTTTTTTTGT TTGCAAGCAG
4801 CAGATTACGC GCAGAAAAAA AGGATCTCAA GAAGATCCTT TGATCTTTTC TACGGGGTCT GACGCTCAGT GGAACGAAAA CTCACGTTAA GGGATTTTGG
4901 TCATGAGATT ATCAAAAAGG ATCTTCACCT AGATCCTTTT AAATTAAAAA TGAAGTTTTA AATCAATCTA AAGTATATAT GAGTAAACTT GGTCTGACAG
5001 TTACCAATGC TTAATCAGTG AGGCACCTAT CTCAGCGATC TGTCTATTTC GTTCATCCAT AGTTGCCTGA CTCCCCGTCG TGTAGATAAC TACGATACGG
bla

5101 GAGGGCTTAC CATCTGGCCC CAGTGCTGCA ATGATACCGC GAGACCCACG CTCACCGGCT CCAGATTTAT CAGCAATAAA CCAGCCAGCC GGAAGGGCCG
bla

5201 AGCGCAGAAG TGGTCCTGCA ACTTTATCCG CCTCCATCCA GTCTATTAAT TGTTGCCGGG AAGCTAGAGT AAGTAGTTCG CCAGTTAATA GTTTGCGCAA
bla

5301 CGTTGTTGCC ATTGCTACAG GCATCGTGGT GTCACGCTCG TCGTTTGGTA TGGCTTCATT CAGCTCCGGT TCCCAACGAT CAAGGCGAGT TACATGATCC
bla

5401 CCCATGTTGT GCAAAAAAGC GGTTAGCTCC TTCGGTCCTC CGATCGTTGT CAGAAGTAAG TTGGCCGCAG TGTTATCACT CATGGTTATG GCAGCACTGC
bla

5501 ATAATTCTCT TACTGTCATG CCATCCGTAA GATGCTTTTC TGTGACTGGT GAGTACTCAA CCAAGTCATT CTGAGAATAG TGTATGCGGC GACCGAGTTG
bla

5601 CTCTTGCCCG GCGTCAATAC GGGATAATAC CGCGCCACAT AGCAGAACTT TAAAAGTGCT CATCATTGGA AAACGTTCTT CGGGGCGAAA ACTCTCAAGG
bla

5701 ATCTTACCGC TGTTGAGATC CAGTTCGATG TAACCCACTC GTGCACCCAA CTGATCTTCA GCATCTTTTA CTTTCACCAG CGTTTCTGGG TGAGCAAAAA
bla

5801 CAGGAAGGCA AAATGCCGCA AAAAAGGGAA TAAGGGCGAC ACGGAAATGT TGAATACTCA TACTCTTCCT TTTTCAATAT TATTGAAGCA TTTATCAGGG
bla

5901 TTATTGTCTC ATGAGCGGAT ACATATTTGA ATGTATTTAG AAAAATAAAC AAATAGGGGT TCCGCGCACA TTTCCCCGAA AAGTGCCACC TGACGTCTAA
6001 GAAACCATTA TTATCATGAC ATTAACCTAT AAAAATAGGC GTATCACGAG GCCCTTTCGT C

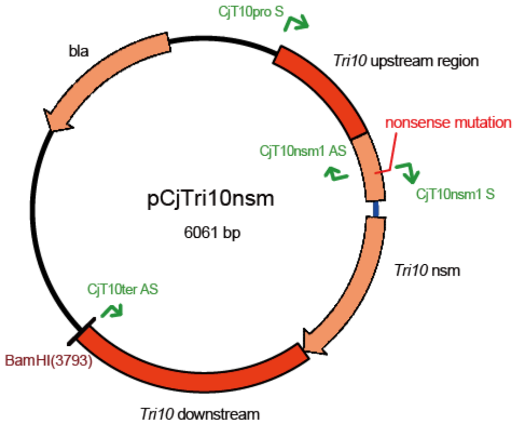

Supplementary Figure 9 | Sequences and structures of pNeotk-SP, pdjTri10tkneo, and pCjTri10nsm. For the construction of pdjTri10tkneo, the *Tri10* locus (*Tri10* and its flanking regions) was amplified with primers DjTri10_NheI_S (5′-TATGCTAGCAGGCGAGTTTGGAAGTATGT -3′) and DjTri10_NheI_AS (5′-ATAGCTAGCCACTCCGTCACAACATAGA A -3′) and digested with *Nhe*I (underlined). After self-ligation of the digested fragment, the circular DNA was used as the template for inverse PCR (Akiyama et al., 2000) with outward primers DjTri10inv_SpeI_AS (5′- GCGACTAGTATCCATGATGACTAACGACA -3′) and DjTri10inv_SacII_S (5′- TACCGCGGATGGTATCGGCGCAGAGA -3′) and digested with *Spe*I (doubly underlined) and *Sac*II (wavy underlined). The *Spe*I - *Sac*II fragment was then cloned into the corresponding sites of pNeotk-SP. The resulting vector, pdjTri10tkneo, was used for transformation of the wild-type strain JCM 9873 to generate Δ*Tri10* tk. For the construction of pCjTri10nsm, two pairs of primers (CjT10pro_S: 5′-TCGAGCTCGGTACCCAGGCGAGTTTGGAAGTATGTT -3′ and CjT10nsm1_AS: 5′- CATACCTACGCCTGTTCAGATCTGCAAG -3′; CjT10nsm1_S: 5′- ACAGGCGTAGGTATGGAAGAGAGAGAAGACAT -3′ and CjT10ter_AS: 5′-CTCTAGAGGATCCCCCACTCCGTCACAACATAGATAT -3′; sequences overlapping in pUC19 underlined) and a nonsense mutation for the gene inactivation (doubly underlined), with 15 bp overhangs necessary for Gibson Assembly, were designed as shown in the figure. The two PCR fragments and *Sma*I-linearized pUC19 were assembled by Gibson Assembly. The resulting vector, pCjTri10nsm, was used for transformation of the transgenic strain Δ*Tri10* tk to generate a marker-free self-cloning strain Δ*Tri10*-nsm.

References

Akiyama, K., Watanabe, H., Tsukada, S., and Sasai, H. (2000). A novel method for constructing gene-targeting vectors. *Nucleic Acids Res.* 28, e77-e77. doi:10.1093/nar/28.16.e77

Chen, L., McCormick, S. P., and Hohn, T. M. (2000). Altered regulation of 15-acetyldeoxynivalenol production in *Fusarium graminearum*. *Appl. Environ. Microbiol.* 66, 2062-2065. doi:10.1128/AEM.66.5.2062-2065.2000

Maeda, K., and Ohsato, S. (2017). Molecular genetic characterization of *Fusarium graminearum* genes identified as encoding a precocene II-binding protein. *JSM Mycotoxins* 67, 1-3. doi:10.2520/myco.67-1-3

Nakajima, Y., Akasaka, M., Shiobara, T., Kitou, Y., Maeda, K., Kanamaru, K., et al. (2020). Impact of nitrogen metabolism-associated culture pH changes on regulation of *Fusarium* trichothecene biosynthesis: revision of roles of polyamine agmatine and transcription factor AreA. *Curr. Genet.* 66, 1179-1190. doi:10.1007/s00294-020-01102-x

Nakajima, Y., Tokai, T., Maeda, K., Tanaka, A., Takahashi-Ando, N., Kanamaru, K., et al. (2014). A set of heterologous promoters useful for investigating gene functions in *Fusarium graminearum*. *JSM Mycotoxins* 64, 147-152. doi:10.2520/myco.64.147

Proctor, R. H., Hohn, T. M., McCormick, S. P., and Desjardins, A. E. (1995). *Tri6* encodes an unusual zinc finger protein involved in regulation of trichothecene biosynthesis in *Fusarium sporotrichioides*. *Appl. Environ. Microbiol.* 61, 1923-1930. doi:10.1128/aem.61.5.1923-1930.1995

Seong, K.-Y., Pasquali, M., Zhou, X., Song, J., Hilburn, K., McCormick, S., et al. (2009). Global gene regulation by *Fusarium* transcription factors *Tri6* and *Tri10* reveals adaptations for toxin biosynthesis. *Mol. Microbiol.* 72, 354-367.

doi:10.1111/j.1365-2958.2009.06649.x

Tag, A. G., Garifullina, G. F., Peplow, A. W., Ake, C., Phillips, T. D., Hohn, T. M., et al. (2001). A novel regulatory gene, *Tri10*, controls trichothecene toxin production and gene expression. *Appl. Environ. Microbiol.* 67, 5294-5302. doi:10.1128/AEM.67.11.5294-5302.2001
